# Assessing water matrix influence and toxicity reduction of crystal violet and reactive black 5 dyes after cold plasma-driven degradation

**DOI:** 10.1038/s41598-026-47084-7

**Published:** 2026-05-04

**Authors:** Shikha Pandey, Ritesh Mishra, Devendra Tiwari, Abhijit Mishra, Sushma Jangra, Indranil Banerjee, Ram Prakash

**Affiliations:** 1 Department of Physics, Indian Institute of Technology Jodhpur, Jodhpur, Rajasthan 342030 India; 2 Inter-Disciplinary Research Division, Smart Healthcare, Indian Institute of Technology Jodhpur, Jodhpur, Rajasthan 342030 India; 3 Department of Bioscience and Bioengineering, Indian Institute of Technology Jodhpur, Jodhpur, Rajasthan 342030 India

**Keywords:** Dielectric barrier discharge, Phytotoxicity, Cytotoxicity, Surface-enhanced Raman spectroscopy, Plasma-activated water, Chemistry, Environmental sciences

## Abstract

Crystal Violet (CV) and Reactive Black 5 (RB5), representative of the triphenylmethane and azo dye classes, are extensively used in the textile industry and are recognized as major contributors to aquatic pollution. Their structural stability and resistance to biodegradation lead to environmental persistence and potential toxicity. In this paper, a non-equilibrium cold plasma-based pin-to-plate dielectric barrier discharge (P2PDBD) source was employed for the degradation of dye solutions. The effects of plasma discharge power, dye concentration, and the chemical composition of the water matrix were systematically studied. With increasing salt concentration from 0 to 25 mg L^−1^, Sodium carbonate synergistically enhanced CV degradation to near completion within minutes but inhibited RB5 by 38%, while sodium chloride, sulfate, and nitrate reduced CV degradation by 16%, 11%, and 8.26%, and RB5 degradation by 39%, 8.36%, and 7.7%, respectively. Radical scavenging experiments using methanol and isopropyl alcohol confirmed that hydroxyl radicals played a dominant role in the degradation process. The initial pH also affected degradation, with CV degrading more rapidly under basic conditions, while RB5 showed reduced efficiencies under both acidic and basic environments. Structural changes and degradation pathways were further elucidated through high-resolution mass spectroscopy, UV–Vis spectroscopy, and surface-enhanced Raman spectroscopy. Post-treatment phytotoxicity and cytotoxicity evaluations indicated that the treated water was non-toxic and, under controlled conditions, may also support early plant growth due to the presence of nitrates, suggesting that plasma-treated effluents are safe and suitable for reuse in agricultural and environmental applications.

**Supplementary Information:**

The online version contains supplementary material available at 10.1038/s41598-026-47084-7.

## Introduction

Most industrial processes use different types of organic dyes to color their products, and the resulting wastewater is often discharged into freshwater bodies such as rivers and streams^1^. These dyes persist in the environment for long periods, posing serious threats to almost all forms of life and disturbing the natural ecological balance^2–4^. Crystal Violet (CV), a triphenylmethane dye, and Reactive Black 5 (RB5), an azo dye, are widely used in the textile industry due to their bright colors and high chemical stability^5,6^. However, their complex molecular structures and resistance to biodegradation make them highly persistent in aquatic environments, leading to severe water contamination. CV has been reported to exhibit genotoxic and mutagenic effects^7^, RB5 is associated with toxic and carcinogenic risks due to the presence of aromatic amine groups in its structure^8^, highlighting the urgent need for their effective removal from wastewater to protect water quality and human health.

Different Advanced Oxidation Processes (AOPs) such as ultrasound^9^, photocatalysis^2,10^, Fenton oxidation^7^, ozone treatment^11^, UV-PCO^12^, cold plasma^13^, catalytic processes^14^ and synergistic AOPs^5,7,14^ have been widely employed for the degradation of CV and RB5. Recently, combined AOPs methods have attracted growing interest because they can deliver higher degradation efficiencies than single-process methods. Chen et al.^5^ demonstrated that incorporating BiPO_4_ photocatalyst into a DBD-driven plasma system led to greater removal of Crystal Violet compared with plasma treatment by itself. Nevertheless, the practical use of catalyst-assisted plasma systems can raise concerns related to catalyst dosage, recovery, reuse, and potential secondary pollution. To avoid these limitations, the present study investigates dye degradation using an independent cold plasma source without the addition of any external catalyst. Cold plasma treatment as a standalone process has been widely reported as an effective approach for the degradation of various dyes in water^15–17^. During plasma discharge, a broad spectrum of highly reactive oxygen species (ROS) is generated, including hydroxyl radicals (^·^OH), ozone (O_3_), singlet oxygen (1O_2_), hydroperoxyl radicals (HO_2_^·^), and hydrogen peroxide (H_2_O_2_)^18^. In addition to these chemical reactions, several physical phenomena such as localized heating, ultraviolet irradiation, and shock wave formation also occur within the discharge region^19,20^. The combined action of these chemical and physical processes enables plasma to efficiently degrade complex dye molecules in aqueous systems without causing secondary pollution. Moreover, after complete dye degradation, the treated solution will not only be free from pollutants but also can be enriched with long-lived reactive species such as nitrates, nitrites, and hydrogen peroxide, transforming it into plasma-activated water (PAW). These species have been widely reported as environmental friendly alternatives to conventional fertilizers like urea, offering additional value for agricultural applications^18,21^. This dual benefit of pollutant removal and the generation of agriculturally useful species is unique to plasma treatment and cannot be achieved by conventional dye removal methods, which only yield clean water devoid of such beneficial reactive species.

Although plasma-based dye degradation has been widely studied, most studies use single-dye solutions in pure water, which do not represent real wastewater conditions^3,22^. Real industrial wastewaters contain various inorganic ions (such as chloride, sulfate, and nitrate) and organic substances that can significantly influence degradation efficiency. The interactions between these ions and plasma-generated reactive species are still not clearly understood. To address these gaps, the present study investigates the degradation mechanisms of structurally different dyes, namely CV and RB5, using the developed P2PDBD plasma source^15^. The influence of water matrix components such as ions, pH, and organic scavenging on degradation efficiency and mechanism is systematically investigated. Degradation intermediates and reaction pathways are analyzed to elucidate the underlying chemistry and environmental implications. The toxicological evaluation of PAW during the process and its byproducts confirms the ecological safety of the process. The findings offer valuable insights for optimizing plasma systems toward practical, low-impact wastewater remediation.

## Materials and methods

### Materials

Commercial RB5 and CV were chosen as model dyes to closely simulate real wastewater scenarios and enhance the relevance of the experimental setup to environmental contexts. Table 1 presents the molecular formula, mass, absorption wavelength, chemical class, initial pH values, molecular structure, and UV–visible spectra of the dyes. All solutions were prepared in DI water, which had a conductivity of 1.6 µS cm^−1^ and a pH of 6.95. Sodium chloride (NaCl), sodium carbonate (Na_2_CO_3_), sodium nitrate (NaNO_3_), sodium sulphate (Na_2_SO_4_), calcium carbonate (CaCO_3_)_,_ and other chemicals used in the experiments were of analytical grade.

**Table 1:** Properties of the dyes used in this study.

Colour index name	RB5	CV
Molecular formula	C_26_H_21_N_5_Na_4_O_19_S_6_	C_25_H_30_ClN_3_
Molecular mass (g mol^−1^)	991.82	407.98
λ_max_ (nm)	598	590
Chemical class	azo dye	triarylmethane dye
Initial pH	6.32	5.54
UV–Vis spectra and molecular structure		

### Plasma source and setup

**Fig. 1:**
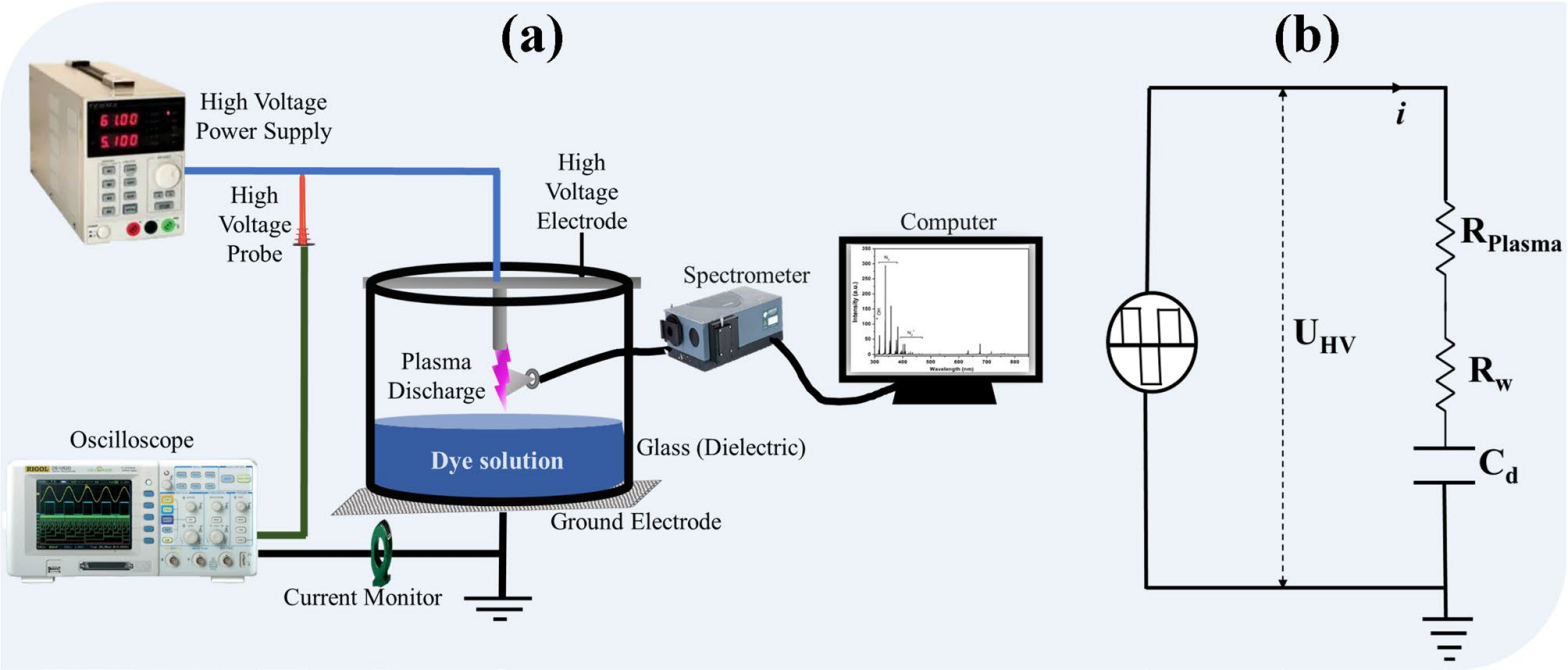
(**a**) Schematic diagram of the Experimental setup for dye solution treatment and (**b**) Electrical circuit diagram of the P2PDBD-based plasma source.

The experimental setup utilized a P2PDBD-based plasma source for the treatment of the dye solution^15^, as depicted in Fig. 1. Power was supplied by a bipolar pulsed high-voltage source (1–25 kV, 1 A, 5–50 kHz pulse repetition rate, 2 μs pulse width). A high-voltage probe (Tektronix P6015A, 75 MHz bandwidth) measured the applied voltage, while a Rogowski coil (Pearson 110, 0.1 V/A, 1 Hz–20 MHz, 20 ns usable rise time) monitored the discharge current. Both devices were connected to a digital oscilloscope (Tektronix MDO3014, 100 MHz bandwidth) to record voltage and current signals. The emission spectra from the discharge were captured across a wavelength range of 200–900 nm using an optical emission spectrometer (Andor Shamrock SR-500i-B1).

### Analytical methods

The instantaneous power (*Pi*(*t*)) facilitated by the bipolar pulsed power supply is calculated by Eq. (1)^23^ mentioned below:

1 $$P_{i} \left( t \right) = V_{i} \left( t \right) \cdot I_{i} \left( t \right)$$

where $V_{i} \left( t \right)$ and $I_{i} \left( t \right)$ are the instantaneous voltage and current.

The dye concentrations before and after plasma treatment were measured by a UV–Vis spectrophotometer (Agilent) with the maximum absorbance intensity at 590 nm for CV and 598 nm for RB5 used for further quantification using a standard curve (SI. I). The degradation efficiency (%) was calculated based on Eq. (2).

2 $$\eta = \frac{{C_{0} - C_{t} }}{{C_{0} }} \times 100\%$$

where *η* is degradation efficiency, $C_{0}$ is the initial dye concentration and $C_{t}$ is the dye concentration after a treatment time $t$, respectively.

The energy yield indicated the amount of dye degraded per unit of energy consumed by the DBD plasma sources, Eq. (3).

3 $$Y = \frac{{ Co V\frac{1}{100} \eta }}{Pt}$$

where $Y$ (mg kWh^-1^) is the energy yield, *C*_*o*_ (mg L^-1^) is the initial concentration of the solution, *V(L)* is the solution volume, *P (kW)* is the discharge power, and *t (h)* is the discharge treatment time.

To investigate the roles of plasma-generated species in the degradation of CV and RB5, scavengers were used to trap specific reactive species, with isopropyl alcohol (IPA) and methanol in the range of 0 to 100 µl added to target OH^·^ radicals. Additionally, the pH was adjusted to 2.78, 6.12, and 10.12 using trimethylamine (N(CH_3_)_3_) and acetic acid (CH_3_COOH). Although the solution pH decreases during plasma exposure to the dye solution^24^. The solution was not buffered in our experiments to simulate real-world conditions. To assess the impact of water matrix on dye degradation, experiments were conducted by adding salts like Na_2_CO_3_, NaCl, Na_2_SO_4_, and NaNO_3_ at concentrations between 0 and 25 mg L^−1^, along with 25 mg L^−1^ of CV and 50 mg L^−1^ of RB5dye solutions. All experiments were performed in duplicate and independently repeated at least three times unless specified otherwise.

### Detection of plasma-generated reactive species and physicochemical analysis in the liquid phase

The concentration of H_2_O_2_ in PAW was measured using the titanium oxysulfate (TiOSO_4_) colorimetric method. In this process, H_2_O_2_ interacts with the titanium sulfonate reagent, forming pertitanic acid (H_2_TiO_4_), which exhibits a yellow color with peak absorbance near 405 nm, as represented in Eq. (4) below^25^. The concentration of nitrate (NO_3_^−^) was determined through the UV screening method^26^. OH^·^ radicals were detected using fluorescence spectrometry (Hitachi, FL4500). In this method, the concentration of OH^·^ radicals are determined by detecting 2-hydroxy terephthalic acid (HTA), which exhibits fluorescence emission at 425 nm when excited by UV light at 310 nm^27^. This occurs through the reaction of terephthalic acid (TA) with OH^·^ radicals generated by the plasma, leading to the formation of HTA, as illustrated in Eq. (5) below. Only a small amount of nitrite was observed in some samples because it rapidly reacts with hydrogen peroxide to form nitrate as shown in Eq. (6); therefore, only hydrogen peroxide and nitrate were measured in this study^28,29^.

4 $${\mathrm{TiOSO}}_{4} + {\mathrm{H}}_{2} {\mathrm{O}}_{2} \to {\mathrm{H}}_{2} {\mathrm{TiO}}_{4} + {\mathrm{H}}_{2} {\mathrm{O}}$$

5 $${\mathrm{C}}_{6} {\mathrm{H}}_{4} \left( {{\mathrm{COOH}}} \right)_{2} + {\mathrm{OH}}^{ \cdot } \to {\mathrm{C}}_{6} {\mathrm{H}}_{3} \left( {{\mathrm{OH}}} \right)\left( {{\mathrm{COOH}}} \right)_{2}$$

6 $${\mathrm{H}}_{2} {\mathrm{O}}_{2} + {\mathrm{NO}}_{2}^{ - } \to {\mathrm{NO}}_{3}^{ - } + {\mathrm{H}}_{2} {\mathrm{O}}$$

### Degradation pathways analysis

The intermediate products formed during the degradation of RB5 and CV dyes by treatment with plasma were identified using high-resolution mass spectrometry (Agilent 6500 Q-TOF LC/MS). Furthermore, the complete degradation of the dyes was confirmed through UV–Vis spectroscopy (Agilent) and surface-enhanced Raman spectroscopy (i-Raman Plus spectrometer (BWTEK)). Raman spectra of both plasma-treated and untreated dye solutions were recorded using anodized aluminum-based SERS substrates at 27 °C utilizing a 784 nm excitation source emitted from a He–Ne laser. The laser beam was precisely focused onto the sample through a microscope objective with 20 × magnification to capture accurate spectra.

### Toxicity analysis

#### Cytotoxicity

To evaluate the cytotoxic effects of the degraded dye samples, NIH3T3 (mouse fibroblast cell line) used in this study was procured from National Centre for Cell Science (NCCS), Pune. The impact of the samples on the cell morphology was first checked through phase contrast microscopy. Cytotoxicity was assessed using both the flow cytometry and MTT assay. For all these studies, NIH3T3 cells were cultured in Dulbecco’s Modified Eagle Medium (DMEM) supplemented with 10% fetal bovine serum (Himedia) and 1% penicillin–streptomycin (Himedia). For the experimental setup, the cells were exposed to PAW, crystal violet (CV, 25 mg L^−1^), and reactive black 5 (RB5, 50 mg L^−1^), along with their plasma-treated counterparts, for treatment durations of 5, 10, and 15 min at a plasma discharge power of 32.6 W. As the samples became acidic after plasma treatment, Na_2_CO_3_ (mM) was added to neutralize the pH and avoid any pH-related effects on cell viability. At the time of addition of the samples to the cells, samples were diluted in the cell culture media following a 1:9 ratio(v/v) and incubated for 24 h at 37 °C in a 5% CO_2_ incubator. Control groups included untreated cells in fresh medium, and cells treated with a mixture of DI water and cell culture media (DI: media = 1:9, v/v). For flow cytometry, 4 × 104 cells were seeded in each well of 24-well tissue culture plate whereas for microscopy and MTT, 1X104 cells were plated on each well of a 96 well tissue culture plate. For flow cytometry, after exposure to CV, RB5, and their plasma-treated versions, both adherent and floating cells were collected, pooled, washed and then stained with propidium iodide (10 mM) for 30 min at 37 °C. Flow cytometry (BD Discover) was performed to determine the proportion of PI-positive (non-viable) cells. This study did not involve any human participants or human-derived samples.

MTT assay was performed using MTT assay kit (Himedia) following the manufactures instructions. Absorbance of the resultant solution was recorded at 570 nm using a microplate reader to determine the cytotoxic effects of the dyes and their degradation products. The experiment was done in triplicate.

#### Phytotoxicity test

To determine whether the treated dye solutions could be safely released into the environment, their potential application as fertilizers was evaluated by conducting seed germination tests and observing improved plant growth through a straightforward procedure^18,30,31^. A hundred uniform seeds of Pearl Millet (*Pennisetum glaucum*), obtained from a local market, were placed in a glass beaker and soaked for 12 h in 30 mL of various solutions, including RB5 solution, CV dye solution, DI water, plasma-activated water (PAW, without dye), and plasma-treated CV and RB5 solutions. After this, 5 mL of the respective solution was added daily to support germination. All experiments were conducted under ambient conditions.

## Results and discussions

### Influence of discharge power and initial concentration on degradation efficiency

The input discharge power plays a critical role in dye degradation by influencing both the physical (e.g., UV light, localized high temperatures) and chemical (e.g., O_3_, H_2_O_2_, e_aq_) effects of plasma discharge^32^. To evaluate the performance and sustainability of the plasma-assisted dye degradation process, energy yield and degradation efficiency were calculated to evaluate not only how effectively the dyes are being degraded but also how efficiently the electrical energy is being utilized for practical and sustainable plasma treatment applications.

**Fig. 2:**
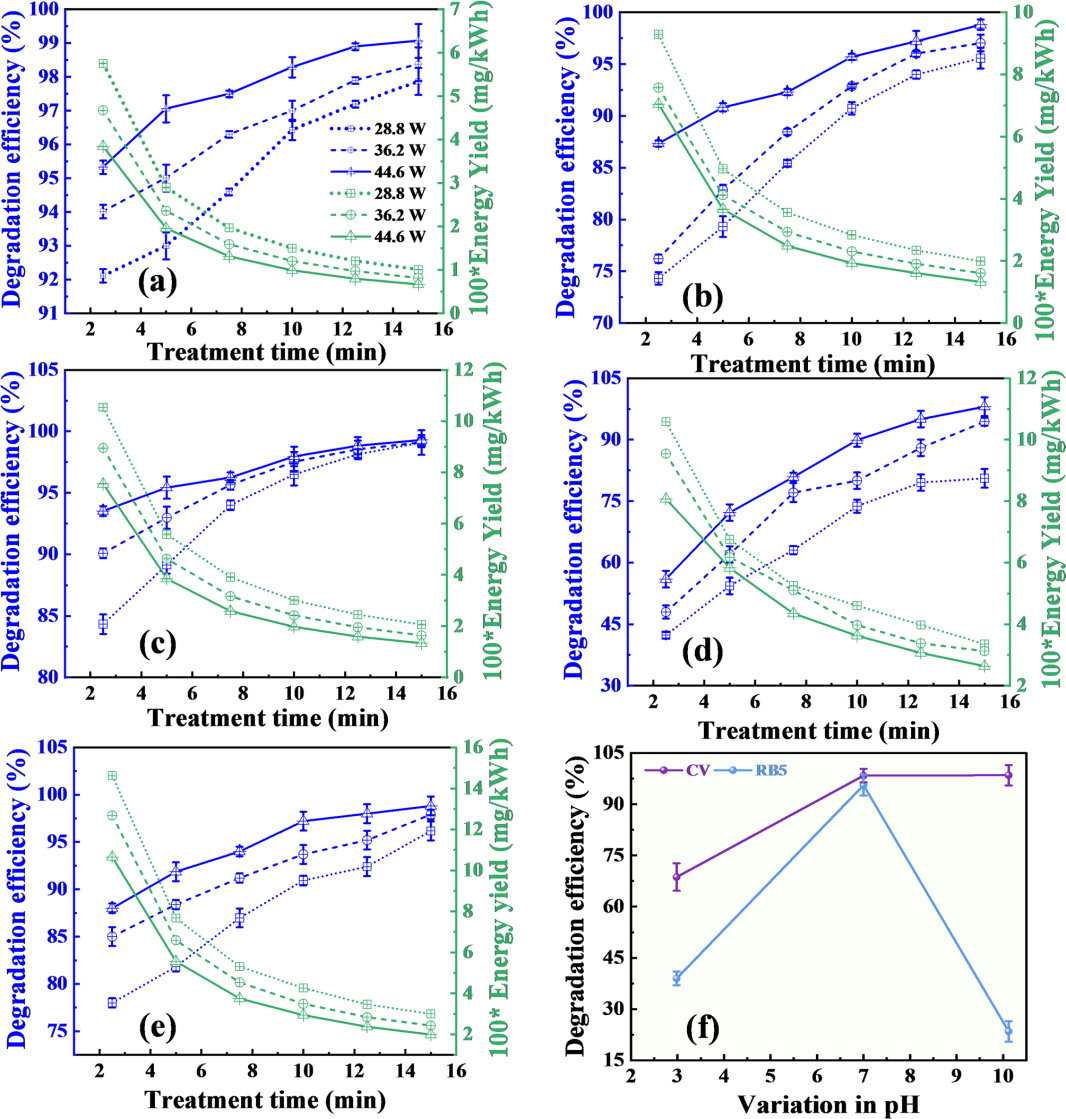
Degradation efficiency and energy yield of (**a**) 25 mg L^−1^ of CV, (**b**) 50 mg L^−1^ of CV, (**c**) 50 mg L^−1^ of RB5, (**d**) 100 mg L^−1^ of RB5, (**e**) mixture of 25 mg L^−1^ of CV and 50 mg L^−1^ of RB5, and (**f**) The influence of pH on the degradation efficiency of CV and RB5 dye at a discharge power of 36.2 W and treatment time of 15 min.

Figure 2a–d depict the results for the mono dyes CV and RB5 under varying conditions of applied power (44.6 W, 36.2 W, and 28.8 W), and discharge time (2.5–15 min). For both dyes, degradation efficiency increases with discharge time as prolonged exposure enhances the cumulative action of plasma-generated reactive species. At higher discharge power (44.6 W), this maximum efficiency is reached more quickly due to the greater density of these reactive species. Interestingly, RB5 consistently demonstrates higher degradation efficiency than CV across all discharge powers, times, and concentrations. This can be explained by structural differences. CV has a triphenylmethane backbone with three bulky, conjugated aromatic rings. These bonds are more stable and require stronger oxidative conditions to cleave. Additionally, steric hindrance makes the approach of reactive species like OH^·^ radicals more difficult. On the other hand, RB5 is an azo dye with relatively weaker azo (–N = N–) bonds, which are more susceptible to attack by radicals and can break down faster^33^. The energy yield exhibits a decreasing trend with increasing discharge time. This decline can be attributed to the saturation effect and the depletion of readily degradable dye molecules, whereby additional energy input results in progressively smaller improvements in degradation performance. Additionally, RB5 shows a higher energy yield compared to CV, reflecting its easier degradation pathway and more efficient utilization of reactive species.

Figure 2e describes the results for the mixture of CV and RB5 dyes. The mixture shows slightly higher degradation efficiency and enhanced energy yield compared to individual dyes. This behavior can be interpreted based on similar principles seen in prior studies on multi-component systems^32,34,35^. In the mixed dye system, both dyes may compete for reactive species, however, their reactivity and complementary degradation pathways enable more efficient utilization of plasma-generated species. The lower initial concentrations of each dye compared to single dye treatments reduce saturation effects and enhance mass transfer and diffusion, leading to improved degradation performance. Furthermore, it can facilitate, indicating the synergistic interactions. These combined effects account for the slightly higher degradation efficiency and energy yield observed in the dye mixture.

Our results show that a moderate power level (36.2 W) provides the optimal balance between dye removal and energy efficiency. Further increasing the power results in only a slight improvement in degradation, while significantly increasing energy consumption and causing additional heating of the plasma source; therefore, the overall benefit becomes limited.

### Influence of pH on degradation efficiency

The textile industry discharges wastewater with a wide range of pH levels^36,37^. Therefore, it is essential to investigate the effect of pH on the efficiency of plasma-based dye degradation. As illustrated in Fig. 2f, the degradation of CV reached nearly 100% at pH 10.12, whereas minimal degradation was observed at pH 2.78. In contrast, RB5 exhibited limited degradation across both acidic and basic pHs. These trends can be explained by the structural variations of the dyes together with the pH-dependent behavior and effective interaction of plasma-generated reactive species under different solution environments. CV has a pKa of 9.4^38^, while RB5 has two key pKa values at 3.8 and 6.9^39^.

At pH 2.78, CV remains in its cationic form, as the pH is significantly lower than its pKa. The strong electrostatic interactions with surrounding water molecules likely hinder its direct reaction with plasma-generated reactive species, leading to slower degradation. The lower degradation efficiency in acidic conditions may also be associated with limited effective interaction between reactive species and the dye molecules^40^. At pH 6.12, CV is still predominantly positively charged, though partial deprotonation may begin. However, due to its high charge density, it maintains strong interactions with water molecules, limiting its availability for oxidative attack and resulting in moderate degradation. In contrast, at pH 10.12, CV undergoes complete deprotonation, reducing its positive charge and electrostatic interactions with water molecules. This transition enhances its hydrophobicity, increasing its susceptibility to hydroxyl radicals, thereby leading to maximum degradation in alkaline conditions^40^. The enhanced degradation at higher pH can be further correlated with improved accessibility of reactive sites and the effective utilization of reactive oxygen species such as OH^·^ and O_2_^−^^33,41^.

RB5, on the other hand, remains largely anionic across the tested pH range due to the presence of sulfonic acid (–SO_3_^−^) groups, which have very low pKa values. At pH 2.78, protonation of the amine (–NH_2_) groups increases the dye’s hydrophilicity and solubility. While this could facilitate interaction with reactive species, RB5 degradation remains limited, likely due to its inherent structural stability and the extended aromatic framework rather than solely radical availability. At pH 6.12, RB5 exists predominantly in its anionic form, maintaining high solubility due to the presence of sulfonate groups. However, degradation remains low, possibly due to steric hindrance and aggregation effects that reduce its exposure to reactive species. At pH 10.12, RB5 remains negatively charged, but it may undergo aggregation in alkaline conditions, further reducing its interaction with hydroxyl radicals. Additionally, the stability of its aromatic rings further restricts its degradation, resulting in lower degradation efficiency at extreme pH levels. Thus, the variability of RB5 degradation with pH is governed more by molecular rigidity and aggregation behavior together with effective reactive species-dye interaction, rather than reactive species concentration alone.

### Influence of water matrix on degradation efficiency

**Fig. 3:**
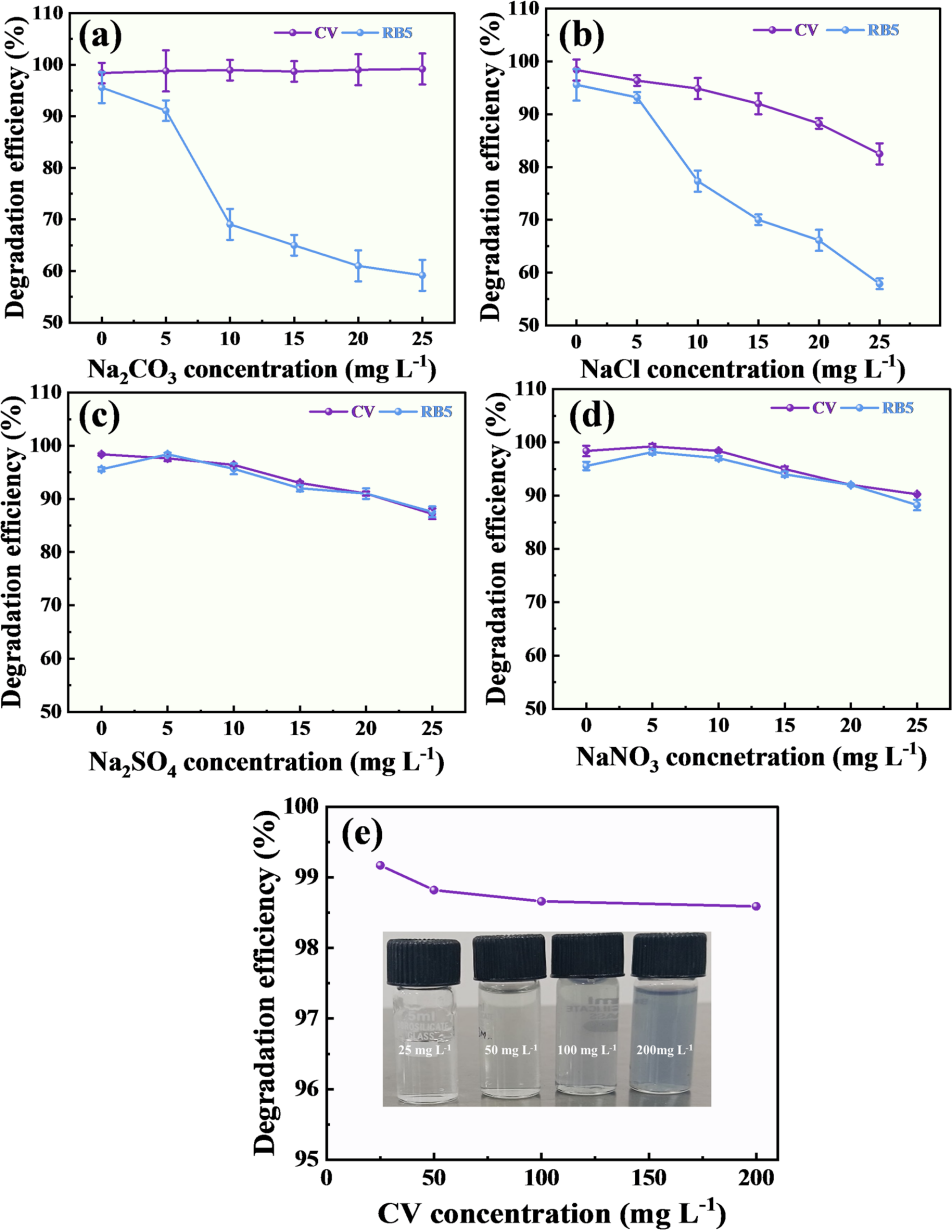
The influence of water matrix on degradation efficiency of CV and RB5 at the power of 36.2 W and treatment time of 15 min (**a**) Na_2_CO_3_, (**b**) NaCl, (**c**) Na_2_SO_4_, (**d**) NaNO_3_, and (**e**) effect of increased concentration of CV at constant 25 mg L^−1^ of sodium carbonate on degradation efficiency.

The substances such as carbonate ions, chloride ions, sulfate ions, nitrate ions, etc., as initial composition in wastewater, are complex and can affect the reactive species composition generated by plasma in the liquid phase, leading to changes in its degradation efficiency. Therefore, the effects of these salts on the degradation efficiency of 25 mg L^-1^ of CV and 50 mg L^-1^ of RB5 dyes are examined. Figure 3a illustrates that the degradation efficiency of CV dye increases with the addition of sodium carbonate, reaching a maximum value of 99.17% at a concentration of 25 mg L^-1^ of sodium carbonate. In contrast, the degradation efficiency of RB5 dye decreases with increasing sodium carbonate concentration, reaching a minimum value of 59.17% at 25 mg L^-1^ of sodium carbonate. This behaviour can be attributed to the shift in pH toward alkaline conditions upon the addition of sodium carbonate, which influences the degradation mechanisms of the dyes, as previously discussed in “Influence of pH on degradation efficiency” section on pH effects^40^. Also, the Carbonate ions (CO_3_^2−^) present in the solution will react with hydroxyl radicals to form carbonate radicals (CO_3_^−·^), expressed below through Eqs. (7) and (8). These carbonate radicals (CO_3_^−·^) are less reactive than OH^·^ but still participate in the dye degradation. The presence of Carbonate ions enhances the overall degradation rate because it doesn’t quench hydroxyl radicals as much as other salts, leaving more OH^·^ available for dye degradation.

7 $${\mathrm{CO}}_{3}^{2 - } + {\mathrm{OH}}^{ \cdot } \to {\mathrm{CO}}_{3}^{ \cdot - } + {\mathrm{H}}_{2} {\mathrm{O}}\quad k = {3}.{9} \times {1}0^{{8}} \;{\mathrm{M}}^{{ - {1}}} \;{\mathrm{S}}^{{ - {1}}}$$

8 $${\mathrm{CO}}_{3}^{ - } + {\mathrm{OH}}^{ \cdot } \to {\mathrm{CO}}_{3}^{ \cdot - } + {\mathrm{OH}}^{ - } \quad k = {3}.{9} \times {1}0^{{8}} \;{\mathrm{M}}^{{ - {1}}} \;{\mathrm{S}}^{{ - {1}}}$$

From Fig. 3b–d, the degradation efficiency of both CV and RB5 dyes decreases with increasing concentration of sodium nitrate, sodium sulfate, and sodium chloride. The addition of these salts will have a minimal effect on the change in the pH of the solution. Therefore, the neutral to slightly acidic conditions are maintained for both CV and RB5, and the effect of the structural variability of CV and RB5 is no longer happening anymore. So, the whole degradation process is dependent only on the availability of plasma reactive species.

The chloride ions (Cl^−^) in the solution react with hydroxyl radicals and form chlorine radicals (Cl^·^) and hypochlorous acid (HOCl), as shown in Eqs. (9) and (10) below. Cl^·^ are strong but selective oxidants, exhibiting high reactivity toward compounds containing aromatic or electron-rich groups. In CV, the electron-donating amine groups (–N(CH_3_)_2_) attached to the aromatic rings increase electron density, which reduces the electrophilic attack by chlorine radicals, making the dye less reactive to Cl^·^. Similarly, RB5, with its azo bonds and electron-rich aromatic rings, is less susceptible to chlorine radical attack. The degradation of RB5 & CV depends on reactive species like OH^·^ radicals or other strong oxidants capable of breaking down its molecular structure. In fact, chloride ions can scavenge hydroxyl radicals, reducing the overall availability of OH^·^ for degrading RB5 & CV. Additionally, chlorine-based reactive species like HOCl or chloramines may form, further decreasing the concentration of hydroxyl radicals available to attack CV and RB5, which ultimately hinders the overall degradation process.

9 $${\mathrm{Cl}}^{ - } + {\mathrm{OH}}^{ \cdot } \to {\mathrm{Cl}}^{ \cdot } + {\mathrm{OH}}^{ - } \quad k = {4}.{3} \times {1}0^{{9}} \;{\mathrm{M}}^{{ - {1}}} \;{\mathrm{S}}^{{ - {1}}}$$

10 $${\mathrm{Cl}}^{ - } + {\mathrm{OH}}^{ \cdot } \to {\mathrm{HOCl}} \quad k = {1}.{3} \times {1}0^{{{10}}} \;{\mathrm{M}}^{{ - {1}}} \;{\mathrm{S}}^{{ - {1}}}$$

The minimal reactivity of sodium nitrate and sodium sulfate with hydroxyl radicals, as shown in Eqs. (11) and (12) below, does not interfere much with degradation but does not enhance it either, leading to moderate degradation of CV and RB5, which is slower degradation compared to Na_2_CO_3_ but potentially faster than NaCl.

11 $${\mathrm{SO}}_{4}^{2 - } + {\mathrm{OH}}^{ \cdot } \to {\mathrm{SO}}_{4}^{ \cdot } + {\mathrm{H}}_{2} {\mathrm{O}}\quad k = {1}.{5} \times {1}0^{{6}} \;{\mathrm{M}}^{{ - {1}}} \;{\mathrm{S}}^{{ - {1}}}$$

12 $${\mathrm{NO}}_{3}^{ - } + {\mathrm{OH}}^{ \cdot } \to {\mathrm{NO}}_{3}^{ \cdot } + {\mathrm{H}}_{2} {\mathrm{O}}\quad k = {1}.{0} \times {1}0^{{5}} \;{\mathrm{M}}^{{ - {1}}} \;{\mathrm{S}}^{{ - {1}}}$$

This suggests that the type of salt present in the solution plays a crucial role in influencing dye degradation behavior. Specifically, sodium carbonate promotes the degradation of CV, likely by facilitating the generation of reactive species, while sodium chloride suppresses the process, possibly due to the scavenging of reactive radicals. Meanwhile, sodium nitrate and sodium sulfate exert minimal influence on CV degradation. In contrast, all the tested salts reduce the removal efficiency of RB5, indicating that the degradation mechanism of this dye is more sensitive to the presence of ionic species.

Figure 3e illustrates that the addition of sodium carbonate markedly enhances the degradation efficiency of CV. When a solution containing 25 mg L^−1^ of CV was treated with an equal concentration (25 mg L^−1^) of sodium carbonate, complete degradation was achieved within 5–7 min of plasma exposure. This result demonstrates a clear synergistic effect between sodium carbonate and cold plasma, allowing nearly 100% degradation in a significantly shorter time under the same discharge power.

To further examine this synergistic interaction, the CV concentration was increased to 200 mg L^−1^ while maintaining the sodium carbonate concentration at 25 mg L^−1^ and keeping it constant. As shown in Fig. 3e, increasing the CV concentration led to a proportional increase in required plasma treatment time, reaching approximately 15 min, for near-complete degradation. These findings indicate that even at lower discharge power, higher concentrations of the persistent CV dye can be effectively degraded through optimized addition of sodium carbonate. This approach not only enhances degradation efficiency but also contributes to energy savings, highlighting its potential for practical wastewater treatment applications.

### Influence of molecular scavenger on dye degradation

**Fig. 4:**
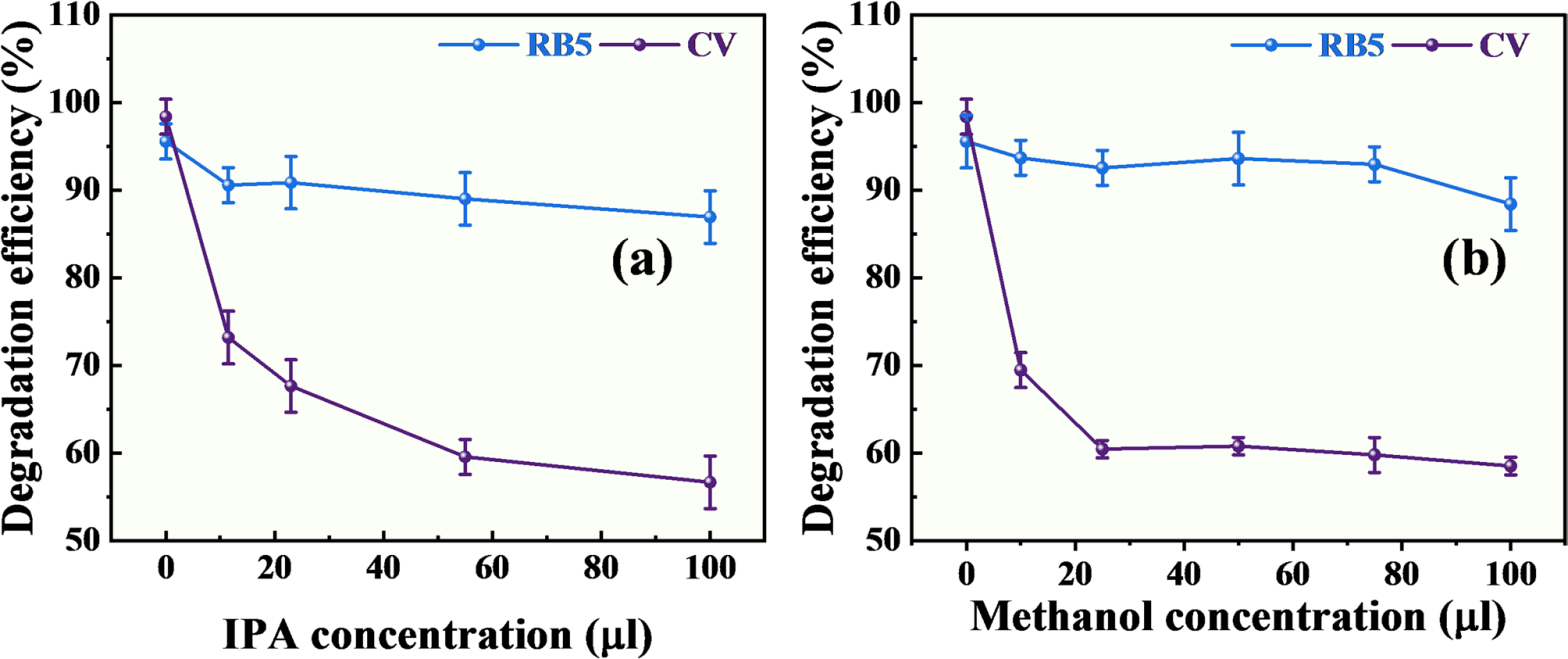
Effect of scavengers on the degradation 25 mg L^−1^ of CV and 50 mg L^−1^ of RB5 dyes (**a**) IPA and (**b**) methanol for a treatment time of 15 min at a discharge power of 36.2 W.

Textile wastewater generally contains various radical-scavenging solvents, such as methanol and IPA, present in varying concentrations^42^. The experimental results, presented in Fig. 4, demonstrate that the presence of scavengers leads to a decrease in degradation efficiency. The plasma source shows a better dye degradation efficiency in the absence of scavengers, achieving almost complete degradation in 15 min at 36.2 W for CV and RB5. The reactions of the scavengers with OH^·^ are given in Eqs. (13) and (14)^23,43^. The variation in reaction times observed with different scavengers can be attributed to their differing reactivity toward OH^·^ radicals^44,45^. The results indicate that methanol has a slower reaction rate with OH^·^, allowing for more efficient degradation of CV and RB5. In contrast, isopropyl alcohol reacts more rapidly with OH^·^, which reduces the availability of these radicals for dye degradation, leading to lower degradation efficiency of CV and RB5.

13 $$2{\mathrm{CH}}_{3} {\mathrm{OH}} + {\mathrm{OH}}^{ \cdot } \to {\mathrm{CH}}_{2} {\mathrm{OH}}^{ \cdot } + {\mathrm{CH}}_{3} {\mathrm{O}}^{ \cdot } + {\mathrm{H}}_{2} {\mathrm{O}}\quad k = {4}.{7} \times {1}0^{{8}} \;{\mathrm{L}}\;{\mathrm{mol}}^{{ - {1}}} \;{\mathrm{s}}$$

14 $$({\mathrm{CH}}_{3} )_{2} {\mathrm{CHOH}} + {\mathrm{OH}}^{ \cdot } \to ({\mathrm{CH}}_{3} )_{2} {\mathrm{COH}}^{ \cdot } + {\mathrm{H}}_{2} {\mathrm{O}}\quad k = {1}.{9} \times {1}0^{{{10}}} \;{\mathrm{L}}\;{\mathrm{mol}}^{{ - {1}}} \;{\mathrm{s}}$$

### Analysis of physicochemical properties of PAW

**Fig. 5:**
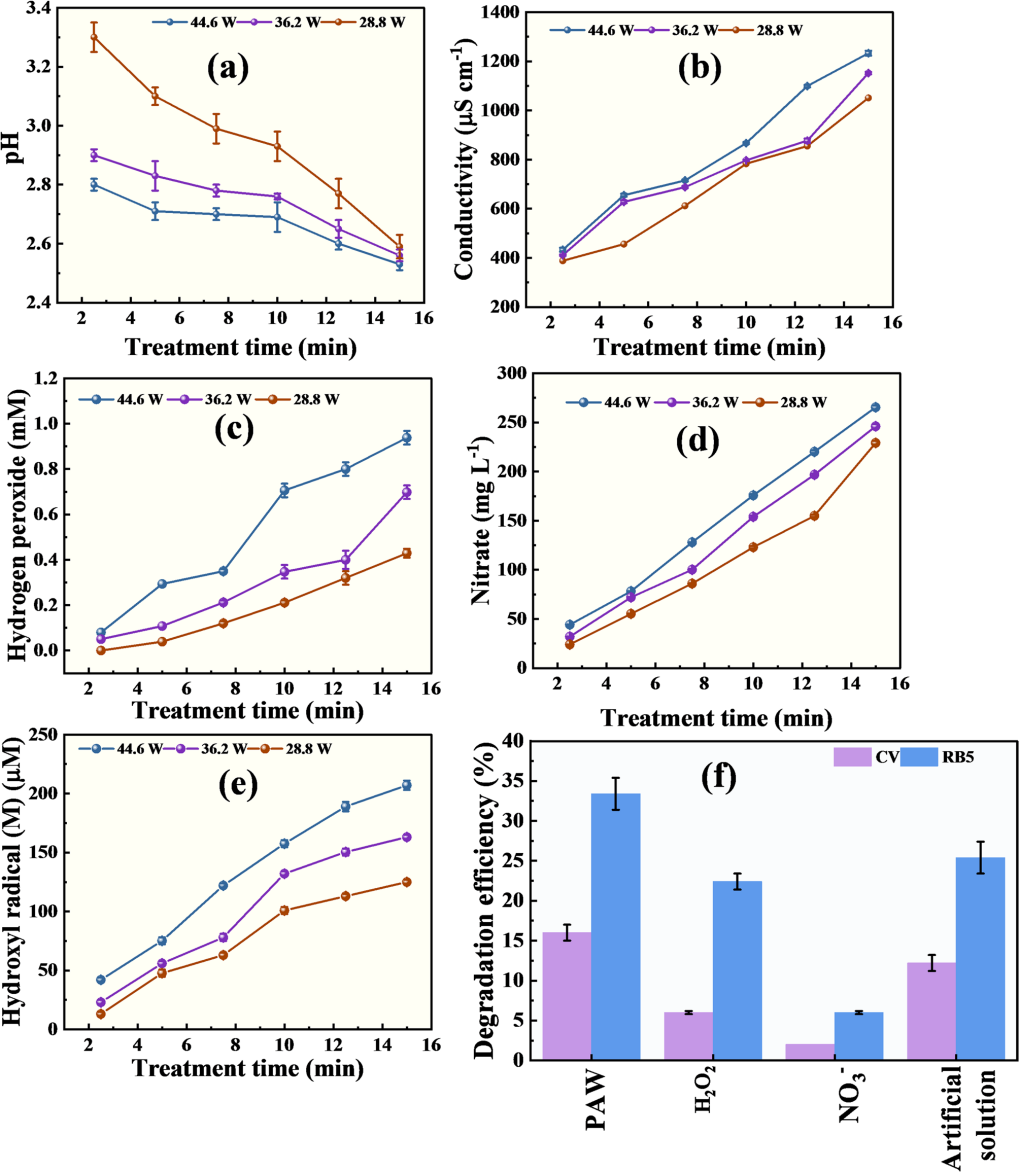
Variation in physicochemical properties of PAW (**a**) pH, (**b**) conductivity, (**c**) hydrogen peroxide, (**d**) nitrate, (**e**) hydroxyl radicals concentrations with plasma treatment time at different discharge powers, and (**f**) Degradation efficiency of CV and RB5 in different samples after 720 min of standing.

As shown in Fig. 5a–e, the physicochemical characteristics of PAW, including pH, conductivity, and concentrations of hydrogen peroxide, nitrate, and hydroxyl radicals, vary with discharge time (2.5–15 min) and applied power (28.8–44.6 W). Since these reactive species likely play distinct roles in dye degradation, their individual contributions were experimentally evaluated*.* For this purpose, PAW was used to prepare aqueous solutions of 25 mg L^-1^ of CV and 50 mg L^-1^ of RB5 dyes, enabling a detailed investigation of plasma-driven degradation pathways in the liquid phase. For ease of discussion, PAW subjected to 15 min of plasma treatment is referred to as PAW15. The generation of RONS results from interactions between plasma constituents formed in air and water molecules at the liquid interface and is described in Eqs. (1)–(13) (see supplementary SI3). As depicted in Fig. 5c, d, treatment at 36.2 W for 15 min produced H_2_O_2_ and NO_3_^−^ concentrations of (0.698 ± 0.03) mM and (246 ± 2) mg L^−1^, respectively.

According to Fig. 5f, the degradation efficiencies of the CV and RB5 solutions prepared with PAW15 reached (16.01 ± 0.98)% and (33.36 ± 0.25)%, respectively, after standing for 720 min. These findings suggest that the reactive species generated in PAW contribute to measurable oxidative activity, leading to gradual dye degradation during prolonged standing. To isolate and assess the specific roles of these species, separate H_2_O_2_ and NO_3_^−^ solutions were prepared at concentrations equivalent to those produced in PAW. Firstly, CV and RB5 dye solutions were prepared by using artificial H_2_O_2_, and allowed to stand for 720 min. The degradation efficiencies obtained in the presence of H_2_O_2_ were (6.01 ± 1.20)% for CV and (22.38 ± 0.22)% for RB5. Secondly, a synthetic NO_3_^−^ solution was used to create the dye solutions for CV and RB5, which were again left to stand for 720 min. In the NO_3_^−^ solution, the degradation efficiencies were (2.06 ± 0.5) % for CV and (6 ± 1.8)% for RB5. To further evaluate the combined influence of these reactive species, an artificial PAW solution containing H_2_O_2_ and NO_3_^−^ at concentrations equivalent to those in PAW was prepared. After 720 min, the degradation efficiencies of 25 mg L^-1^ of CV and 50 mg L^-1^ of RB5 dyes reached (12.24 ± 0.36)% and (25.38 ± 0.28)%, respectively.

These results indicate that among the long-lived reactive species, H_2_O_2_ contributes most significantly to dye degradation. This predominant role can be attributed to its high stability, extended half-life, and its capacity to decompose into highly reactive OH^·^ radicals, which exhibit a reaction rate constant of 7.7 × 10⁷ M^−1^ s^−1^^45^. Additionally, the low pH of PAW further supports the formation of OH^·^ radicals through the decomposition of peroxynitrous acid, as shown in Eq. (15). Hydroxyl radicals can also form via water dissociation at the plasma–liquid interface, as described in Eqs. (15) and (16)^13^.

1. At low pH (< 3.5), peroxynitrous acid (O=NOOH) decomposes to release OH^·^ radicals
  15 $${\mathrm{ONOOH}} \to {\mathrm{OH}}^{ \cdot } + {\mathrm{NO}}_{2}^{ \cdot }$$
2. Water dissociation
  16 $${\mathrm{H}}_{2} {\mathrm{O}} \to {\mathrm{OH}}^{ \cdot } + {\mathrm{H}}^{ \cdot }$$

The convection generated by plasma activity accelerates the migration of dye molecules (e.g., CV and RB5) toward the plasma–liquid interface, where reactive species are concentrated. This enhanced mass transport increases the frequency and efficiency of dye-radical interactions, thereby improving degradation performance^13^. This coordinated interplay of reactive species generation, pH-dependent pathways, and convective transport results in the limited but noticeable degradation of dyes during plasma treatment. The degradation mechanism, primarily driven by hydroxyl radicals, has been further characterized through HRMS, SERS, and UV–vis spectroscopy.

### Degradation analysis of CV and RB5

#### Surface enhanced Raman spectroscopy analysis

**Fig. 6:**
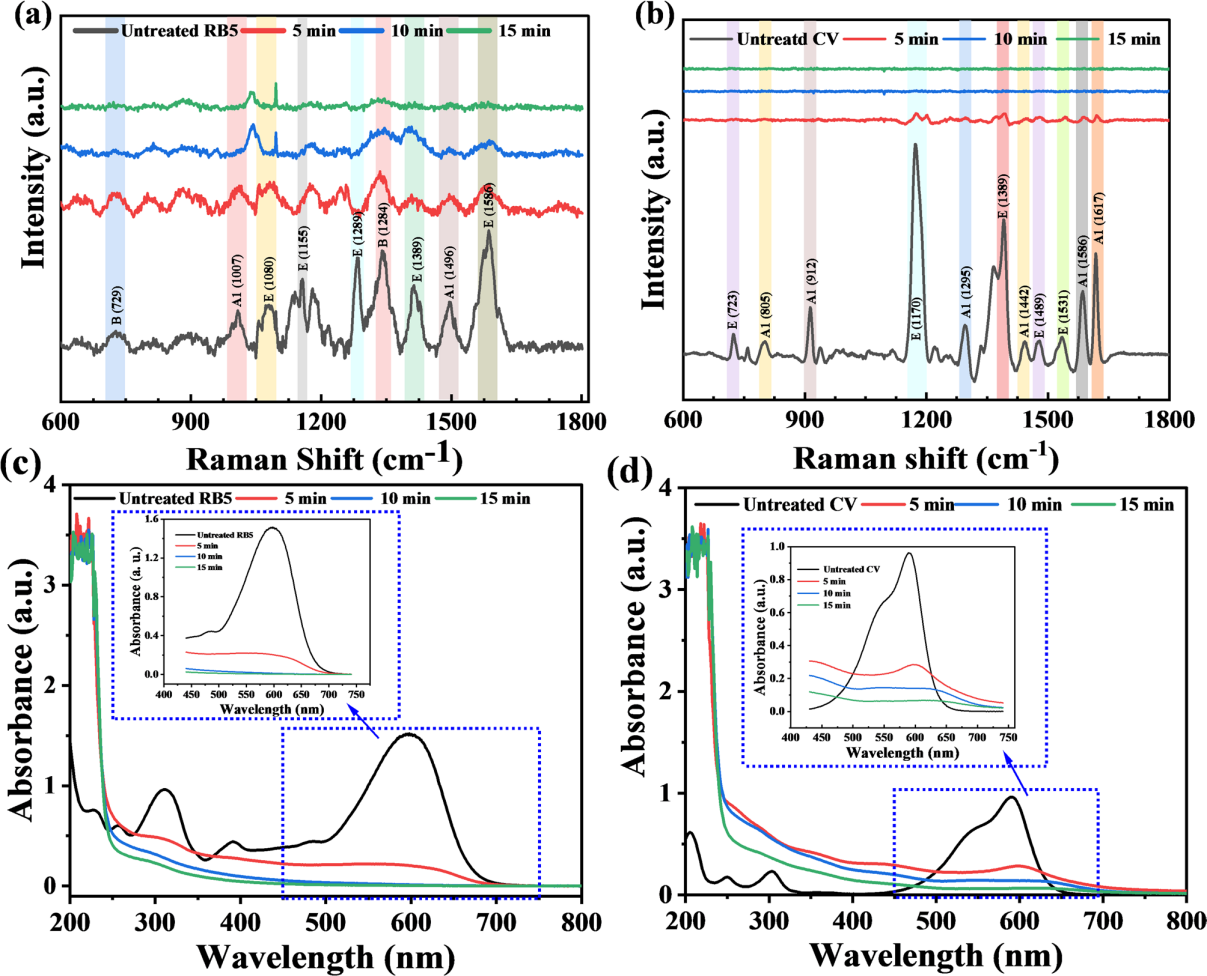
SERS of (**a**) 50 mg L^−1^ of RB5 and (**b**) 25 mg L^−1^ of CV & UV–visible spectra of (**c**) 50 mg L^−1^ of RB5 and (**d**) 25 mg L^−1^ of CV at different treatment times at a discharge power of 36.2 W.

SERS of RB5 and CV provide valuable insights into the structural integrity of both dyes and the impact of plasma treatment on their degradation pathways. For RB5, characteristic peaks corresponding to its azo (–N=N–) bonds, aromatic rings (benzene and naphthalene), and sulfonate (–SO_3_^−^) functional groups highlight key molecular interactions. Notably, the B_2_g mode at 729 cm^−1^ (C–S stretching), A_1_g mode at 1007 cm^−1^ (benzene and naphthalene ring breathing), and E_1_g mode at 1080 cm^−1^ (C–H bending with sulfonate stretching) reflect RB5’s structural stability. Peaks at 1342 cm^−1^ (N=N symmetric stretching), 1499 cm^−1^ (N=N asymmetric stretching), and 1586 cm^−1^ (C=C aromatic stretching) are critical indicators of chromophoric properties^46^. Under plasma treatment, significant spectral modifications occur, including the disappearance of these peaks, indicating cleavage of azo bonds, disruption of aromatic conjugation, and degradation of sulfonate groups, as shown in Fig. 6a. These changes confirm that plasma-based degradation effectively breaks down RB5’s structural framework, leading to decolorization and molecular fragmentation.

Similarly, the SERS spectrum of CV reveals vibrational peaks characteristic of its triphenylmethane structure. The E_1_g mode at 723 cm^−1^ (C–N–C ring bending), A_1_g mode at 805 cm^−1^ (out-of-plane C–H bending), and A_1_g mode at 912 cm^−1^ (ring breathing mode) highlight skeletal deformations. Higher-wavenumber peaks, such as the A_1_ mode at 1295 cm^−1^ (C–N stretching) and E mode at 1390 cm^−1^ (aromatic C–C stretching), play a role in CV’s electronic properties and chromophoric stability^47^. With increasing plasma treatment time, the progressive disappearance of peaks at 1619 cm^−1^, 1586 cm^−1^ (C=C stretching), 1477 cm^−1^ (C–C stretching), and 1531 cm^−1^ (C–N symmetric stretching) suggests decomposition of the triphenylmethane core and oxidation of aromatic rings. The broadening and attenuation of peaks such as 1295 cm^−1^ and 1390 cm^−1^ further indicate C–N and C–C bond modifications, leading to fragmentation and degradation, as shown in Fig. 6b. The comparative SERS analysis of RB5 and CV under plasma treatment confirms a common degradation mechanism involving the cleavage of conjugated bonds, oxidation of aromatic rings, and disruption of functional groups critical to dye stability.

#### UV–visible spectroscopy analysis

The UV–visible spectra of treated and untreated CV and RB5, presented in Fig. 6c, d, corroborate the findings observed in the SERS shown in Fig. 6a, b. As illustrated in Fig. 6c, d, the degradation of both RB5 and CV is clearly evident. A progressive decline in the absorbance intensities of the characteristic peaks associated with RB5 and CV is observed with increasing treatment time, signifying the effective degradation of both dyes over the course of the plasma treatment, which is in agreement with the reported work^3,12^. Additionally, an increase in absorbance within the wavelength range of (200–400) nm with prolonged plasma treatment indicates the presence of RONS generated in the water qualitatively^18,48^. This observation suggests the formation of RONS due to plasma exposure, which likely contributes to the dye degradation process.

### Toxicological tests of PAW

#### Cytotoxicity test

Persistence of synthetic dyes in the environmental poses moderate to severe risks to human health^49^. One of the preliminary approaches to assess this risk is to check the cytotoxicity in vitro. Here we test the toxicity of the degraded dye samples on normal cells (NIH3T3) to have an idea about the potential health risk.

**Fig. 7:**
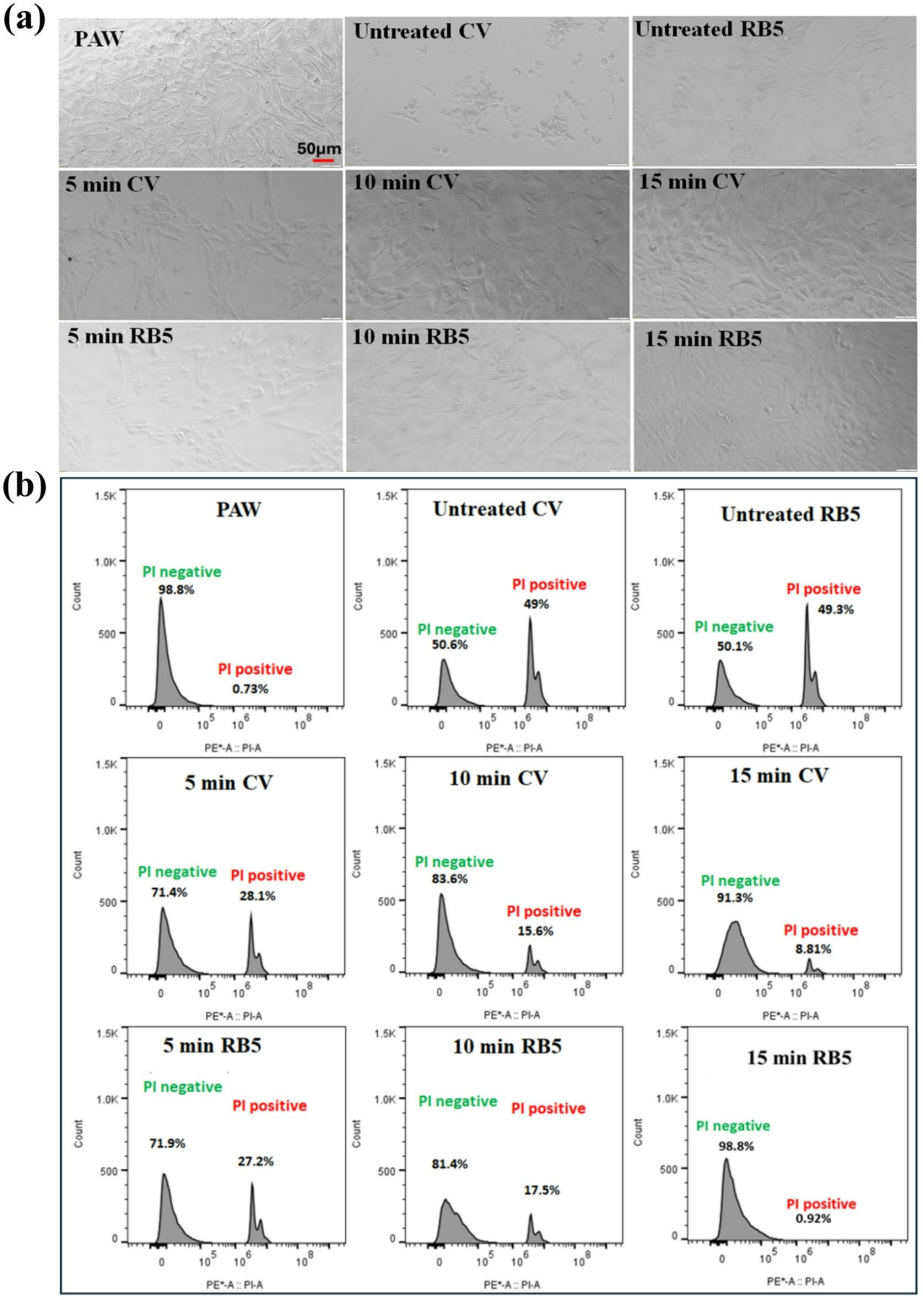
(**a**) Phase-contrast microscopy images of NIH3T3 mouse fibroblast cells, (**b**) quantification of PI-positive (dead) cells using flow cytometry for untreated and plasma-treated CV and RB5 dyes.

The Fig. 7 shows the phase-contrast microscopy and flow cytometry results comparing the effects of untreated and plasma-treated dyes CV and RB5 on cell viability. Phase-contrast microscopy images, shown in Fig. 7a, provide a comparative idea about the morphological changes in NIH3T3 mouse fibroblast cells upon exposure to untreated and plasma-treated dyes in aqueous solution. The control group (cells treated with media) exhibited a healthy, confluent monolayer, indicating normal cell growth. Similarly, cells treated with media + DI Water (9:1, v/v) and PAW showed no significant alterations in morphology, confirming that exposure PAW alone did not induce cytotoxic effects (Supplementary section II). In contrast, cells exposed to untreated CV and RB5 dye solution displayed considerable structural damage, characterised by cell shrinkage and detachment, suggesting that both dyes, in their untreated forms, exert cytotoxic effects on NIH3T3 cells.

However, plasma-treated dye solutions showed a noticeable improvement in cell morphology compared to their untreated counterparts. With increasing plasma treatment time (5, 10, and 15 min), cellular response varied. Cells in the 5-min plasma treatment showed sign of cell damage however the extent was found comparatively less in comparison to the cells treated with dye solution (without plasma treatment), indicating a possible reduction in toxicity. With further increase in plasma treatment time (10 min CV, 15 min CV, 10 min RB5, and 15 min RB5), visual sign of cellular damage was found progressively disappearing, suggesting that plasma treatment effectively degraded the toxic components of the dyes. The overall trend in the microscopy images suggests that untreated dyes significantly damage cell viability, while plasma-treated dyes, especially at longer treatment durations, lead to reduced toxicity.

For the quantitative study, flow cytometry (Fig. 7b) was performed to assess cell viability. For this purpose, PI was used as a marker for dead cells. Study showed that control samples, (cells treated with media and cells treated with media + DI Water (9:1, v/v) have minimal PI positive cell population (0.43–0.88%), confirming high viability (Supplementary section). Similarly, it was observed that PAW induced only ~ 0.73% cell death, indicating negligible cytotoxicity. Untreated dyes (CV & RB5), by contrast, caused substantial cell death, with ~ 49% and ~ 49.5% of cells found PI-positive, respectively, indicating significant membrane damage. Interestingly, it was observed that plasma treatment progressively reduced this toxicity. For ‘5 min CV’, PI-positive cell populations were found to be 28.1% whereas the same was found to be 8.81%for ‘15 min CV’. A similar trend was also found for plasma-treated RB5. In the case of RB5, the PI-positive cell population was 27.2% for ‘5 min RB5’, whereas the same was 0.92% for 15 min RB5, demonstrating near-complete detoxification. These observations are consistent with phase-contrast microscopy. The cytotoxicity assay was also tallied with MTT assay results (supplementary section), confirming that plasma treatment effectively reduces dye-induced cytotoxicity.

These results indicate a reduction in cytotoxicity after plasma treatment of dye solutions. However, testing on additional cell lines and in-vivo models would be required for a more complete biological safety evaluation.

#### Phytotoxicity test

**Fig. 8:**
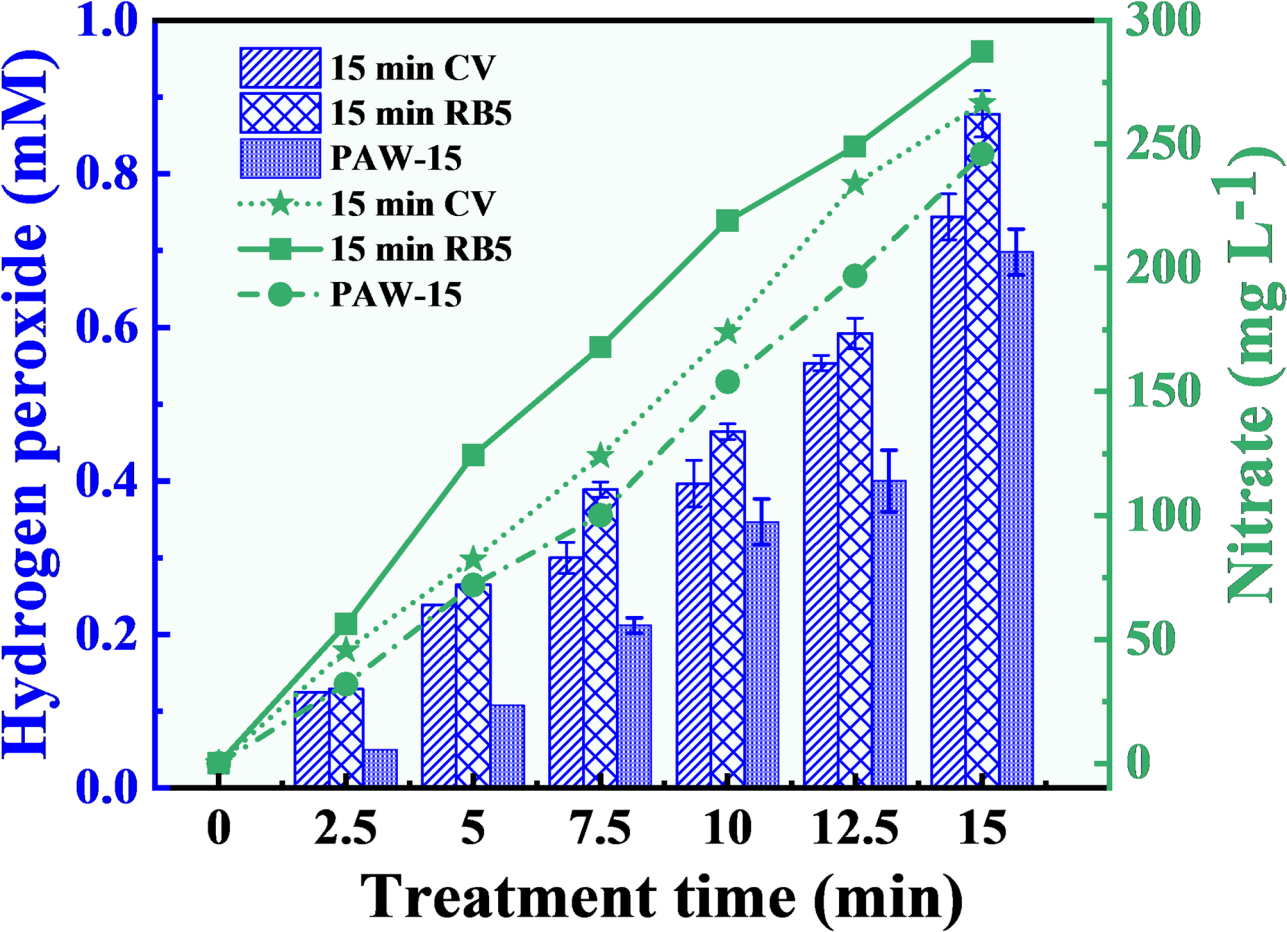
The concentration of nitrate and hydrogen peroxide in plasma-treated CV and RB5 solutions at 36.2 W discharge power.

The concentrations of NO_3_^−^ ions in PAW-15, plasma-treated CV, and RB5 were measured as (246 ± 2.2) mg L^−1^, (266.48 ± 1.2) mg L^−1^, and (287.43 ± 0.22) mg L^−1^, respectively (see Fig. 8). The higher nitrate concentration observed during RB5 degradation compared to CV is attributed to its chemical structure, which contains two azo (–N=N–) bonds and amine (-NH_2_) groups that are susceptible to oxidation to NO_3_^−^ under plasma treatment^41^. In contrast, CV, which lacks azo bonds, generates nitrate predominantly through the oxidation of its tertiary amine (–N(CH_3_)_2_) groups.

**Fig. 9:**
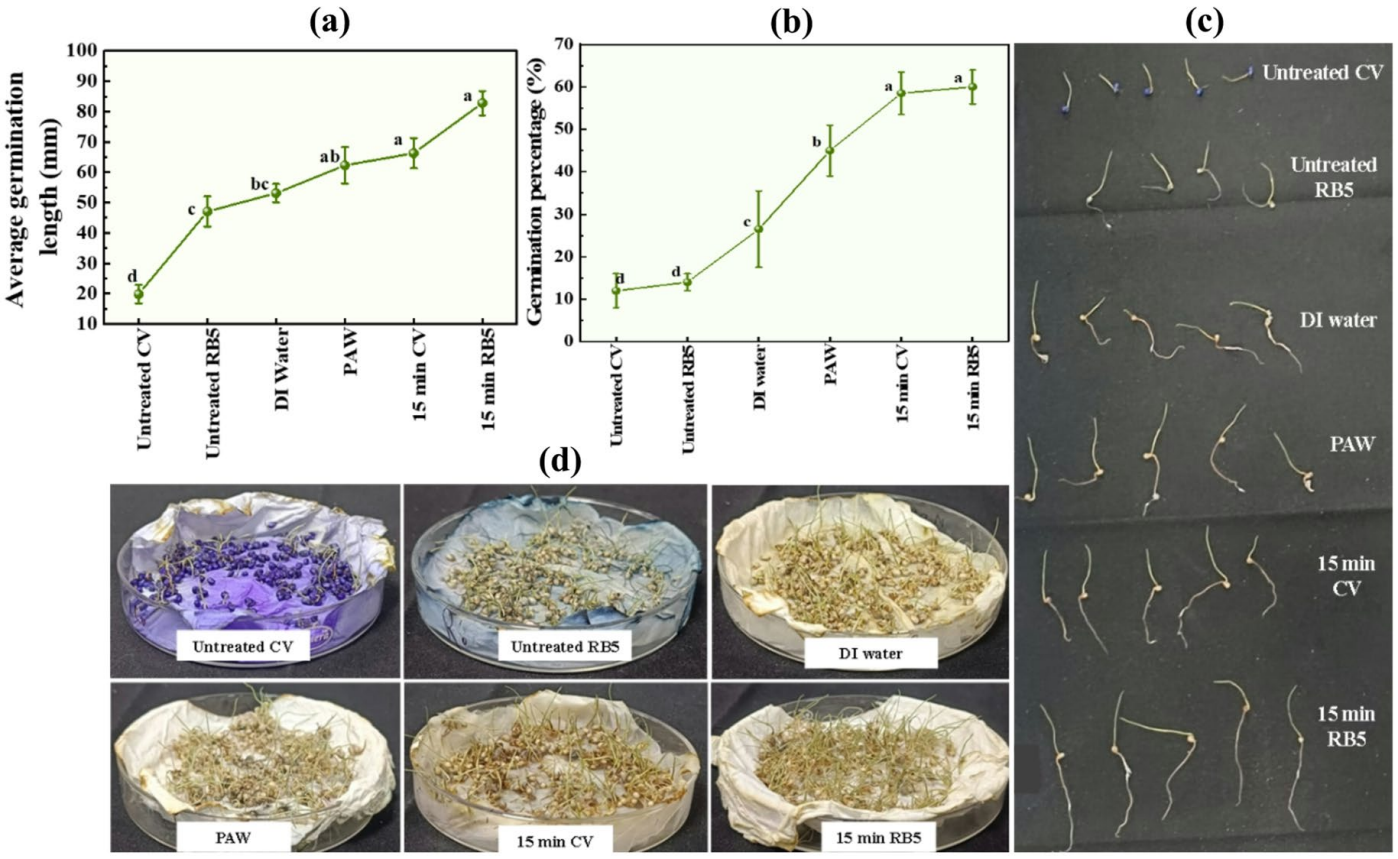
(**a**) Line graphs of average germination length, (**b**) germination percentage, and (**c**, **d**) photographs of shoot and root lengths of pearl millet seeds over 4 days.

The effects of PAW and degraded dye solutions, on seed germination and early plant growth are also evaluated. Treatments with PAW, as well as 15-min plasma-treated CV and RB5 solutions, resulted in seed germination percentages of (45 ± 6)%, (58.5 ± 5)%, and (60 ± 4)%, respectively, within 48 h (see Fig. 9). In contrast, seeds irrigated with deionized (DI) water, untreated CV, and RB5 exhibited germination rates of only (26.5 ± 9)%, (12 ± 4)%, and (14 ± 2)%, respectively. Furthermore, after four days of irrigation with the respective solutions (DI water, dyes, PAW, and plasma-treated dye solutions), germination length was measured. As shown in Fig. 9, seeds treated with plasma-degraded dyes demonstrated significantly greater growth compared to all other treatments. These results suggest that plasma-treated dye solutions, which are enriched with NO_3_^−^ and contain moderate levels of H_2_O_2_, have potential for agricultural reuse. Such applications may support plant growth under controlled conditions while simultaneously contributing to the mitigation of wastewater disposal challenges. However, it is important to note that plant responses to plasma-treated water may vary across species due to differences in tolerance to reactive oxygen and nitrogen species. Therefore, further studies are required encompassing a broader range of plant species to assess the generalizability and safety of utilizing plasma-treated dye solutions in agricultural applications.

## Conclusions

The application of cold plasma source for the degradation of CV and RB5 in wastewater offers a sustainable and efficient solution to mitigate pollution caused by these widely used textile dyes. This study demonstrated that the degradation efficiency of CV and RB5 is significantly influenced by discharge power, treatment time, initial dye concentration, solution pH, and the presence of various ions in the water matrix. Carbonate ions are found to promote CV degradation but inhibit that of RB5, while chloride, sulfate, and nitrate ions generally suppress the degradation of both dyes. OH^·^ radicals were confirmed through molecular scavenger studies, are identified as the primary reactive species driving the oxidative breakdown of CV and RB5. The degradation pathways involve the cleavage of azo and aromatic structures, followed by desulfonation and ring-opening reactions, ultimately leading to degradation into non-toxic end products such as CO_2_, SO_4_^2−^, NO_3_^−^, and H_2_O.

High-resolution mass spectrometry, surface-enhanced Raman spectroscopy, and UV–Vis spectroscopy collectively provided in-depth insights into the formation and transformation of intermediate degradation products during plasma treatment. Cytotoxicity and phytotoxicity assays confirmed that appropriately treated CV and RB5 water solutions exhibited no detectable toxicity. Seed germination experiments further demonstrated that plasma-treated water was not only non-toxic but also promoted plant growth, likely due to the presence of nitrates and moderate concentrations of hydrogen peroxide. These findings indicate that plasma-treated, dye-containing wastewater holds promise for reuse in agricultural applications, thereby contributing to both environmental remediation and resource recovery.

The present investigation was conducted under controlled single-ion conditions to systematically elucidate the influence of individual salts on plasma degradation efficiency. While these results provide mechanistic insight, real wastewater streams typically contain multiple coexisting ions and organic constituents that may introduce competitive or synergistic effects. Nevertheless, further research is required to optimize plasma operating parameters and to evaluate the long-term effects on various plant species and soil ecosystems. Overall, this study offers valuable insights and practical guidance for the development of efficient, safe, and sustainable cold plasma-based technologies for dye degradation and wastewater treatment.

## Supplementary Information

Below is the link to the electronic supplementary material.

Supplementary Material 1

## Data Availability

The datasets used and/or analysed during the current study are available from the corresponding author on reasonable request.
