# Supplementary material for: Assessing water matrix influence and toxicity reduction of crystal violet and reactive black 5 dyes after cold plasma-driven degradation
Source: Sci Rep. 2026 May 4;16:20598. doi: 10.1038/s41598-026-47084-7 (PMC13334030; doi:10.1038/s41598-026-47084-7)
Supplement: Supplementary file 1 — Supplementary Material 1 [file 41598_2026_47084_MOESM1_ESM.docx]

**Assessing Water Matrix Influence and Toxicity Reduction of Crystal Violet and Reactive Black 5 Dyes After Cold Plasma-Driven Degradation**

Shikha Pandey^a^, Ritesh Mishra^b^, Devendra Tiwari^c^, Abhijit Mishra^a^, Sushma Jangra^a^, Indranil Banerjee^c^, and Ram Prakash^a,b,*^

^a^Department of Physics, Indian Institute of Technology Jodhpur, Rajasthan 342030,

^b^Inter-Disciplinary Research Division, Smart Healthcare, Indian Institute of Technology Jodhpur, Rajasthan

342030, India

^c^Department of Bioscience, Indian Institute of Technology Jodhpur, Rajasthan 342030,

*Corresponding author: [ramprakash@iitj.ac.in](mailto:ramprakash@iitj.ac.in)

1. **Standard Curves of CV and RB5**

To determine the unknown concentrations of the CV, RB5, and mixture of dyes, standard calibration curves were prepared, as shown in Fig.1 (a,b & c) below.

**Fig.1:** Calibration curves of (a) CV, (b) RB5, (c) mixture of RB5 and CV.

1. **Characterization of a plasma source**

Fig. 2 presents the voltage and current waveforms of the DBD plasma source at applied voltages of 6, 7, and 8 kV. The VI characteristics indicate a filamentary discharge, as shown by distinct current spikes. The power consumed for 6, 7, and 8 kV is (28.8 ± 0.4) W, (36.2 ± 0.2) W, and (44.6 ± 0.5) W, respectively. Air is used as the discharge gas in this study due to its abundance, low cost, and role as a rich source of oxygen- and nitrogen-based reactive species, which are critical for plasma-induced reactions. In comparison, noble gases such as argon or helium are expensive and less practical for continuous or large-scale applications. To identify the key reactive species generated in the air plasma, Optical Emission Spectroscopy (OES) diagnostic was used, and spectra were recorded. As shown in Fig. 2(d), prominent emissions from the nitrogen second positive system (C³Πᵤ → B³Πg) at 297.8, 315.8, 316.3, 337.1, 353.5, 357.6, and 380.4 nm, along with the first negative system of N₂⁺ (B²Σᵤ⁺ → X²Σg⁺) at 391.4, 399.9, and 405.3 nm, were identified. These species are generated by electron impact excitation due to the high concentration of molecular nitrogen in the atmosphere [1,2]. Additionally, the presence of abundant OH^.^ radicals are confirmed by their characteristic emission transitions observed around 306–309 nm, further highlighting the formation of reactive oxygen species in air plasma [3].

**Fig.2** V-I characteristics of P2PDBD-based plasma source at constant frequency 20 kHz and different applied voltages (a) 6 kV, (b) 7 kV, (c) 8 kV, and (d) OES of P2PDBD-based plasma source at constant frequency and voltage, 20 kHz/7 kV.

1. **Analysis of physicochemical properties of PAW**

The generation of RONS results from interactions between plasma constituents formed in air and water molecules at the liquid interface and is described in Equations (1) – (13).

${\boldsymbol{2}\boldsymbol{H}}_{\boldsymbol{2}}\boldsymbol{O}\boldsymbol{+}\boldsymbol{e}^{\boldsymbol{-}}\boldsymbol{\to}\boldsymbol{H}_{\boldsymbol{2}}\boldsymbol{O}_{\boldsymbol{2}}\boldsymbol{+}\boldsymbol{H}_{\boldsymbol{2}}\boldsymbol{+}\boldsymbol{e}^{\boldsymbol{-}}$ **(1)**

$\boldsymbol{H}_{\boldsymbol{2}}\boldsymbol{O}\boldsymbol{+}\boldsymbol{e}^{\boldsymbol{-}}\boldsymbol{\to}\boldsymbol{H}^{\boldsymbol{+}}\boldsymbol{+}\boldsymbol{e}^{\boldsymbol{-}}$ **(2)**

$\boldsymbol{OH}^{\boldsymbol{.}}\boldsymbol{+}\boldsymbol{OH}^{\boldsymbol{.}}\boldsymbol{\to}\boldsymbol{H}_{\boldsymbol{2}}\boldsymbol{O}_{\boldsymbol{2}}$ **(3)**

$\boldsymbol{H}_{\boldsymbol{2}}\boldsymbol{O}\boldsymbol{+}{\boldsymbol{2}\boldsymbol{NO}}_{\boldsymbol{2}}\boldsymbol{\to}{\boldsymbol{2}\boldsymbol{H}}^{\boldsymbol{+}}\boldsymbol{+}\boldsymbol{NO}_{\boldsymbol{2}}^{\boldsymbol{-}}\boldsymbol{+}\boldsymbol{NO}_{\boldsymbol{3}}^{\boldsymbol{-}}$ **(4)**

$\boldsymbol{H}_{\boldsymbol{2}}\boldsymbol{O}\boldsymbol{+}\boldsymbol{NO}_{\boldsymbol{2}}\boldsymbol{+}\boldsymbol{NO}\boldsymbol{\to}{\boldsymbol{2}\boldsymbol{H}}^{\boldsymbol{+}}\boldsymbol{+}{\boldsymbol{2}\boldsymbol{NO}}_{\boldsymbol{2}}^{\boldsymbol{-}}$ **(5)**

$\boldsymbol{O}_{\boldsymbol{3}}\boldsymbol{+}\boldsymbol{NO}_{\boldsymbol{2}}^{\boldsymbol{-}}\boldsymbol{\to}\boldsymbol{O}_{\boldsymbol{2}}\boldsymbol{+}\boldsymbol{NO}_{\boldsymbol{3}}^{\boldsymbol{-}}$ **(6)**

$\boldsymbol{NO}_{\boldsymbol{2}}^{\boldsymbol{-}}\boldsymbol{+}\boldsymbol{NO}_{\boldsymbol{3}}\boldsymbol{\to}\boldsymbol{NO}_{\boldsymbol{2}}\boldsymbol{+}\boldsymbol{NO}_{\boldsymbol{3}}^{\boldsymbol{-}}$ **(7)**

$\boldsymbol{NO}_{\boldsymbol{2}}^{\boldsymbol{-}}\boldsymbol{+}\boldsymbol{H}^{\boldsymbol{+}}\boldsymbol{+}\boldsymbol{H}_{\boldsymbol{2}}\boldsymbol{O}_{\boldsymbol{2}}\boldsymbol{\to}{\boldsymbol{ONOOH}\boldsymbol{+}\boldsymbol{H}}_{\boldsymbol{2}}\boldsymbol{O}$ **(8)**

$\boldsymbol{O}_{\boldsymbol{3}}\boldsymbol{+}\boldsymbol{UV}\boldsymbol{\to}\boldsymbol{O}_{\boldsymbol{2}}\boldsymbol{+}\boldsymbol{O}^{\boldsymbol{.}}$ **(9)**

$\boldsymbol{H}_{\boldsymbol{2}}\boldsymbol{O}\boldsymbol{+}\boldsymbol{O}^{\boldsymbol{.}}\boldsymbol{\to}{\boldsymbol{2}\boldsymbol{OH}}^{\boldsymbol{.}}$ **(10)**

$\boldsymbol{O}_{\boldsymbol{3}}\boldsymbol{+}\boldsymbol{H}_{\boldsymbol{2}}\boldsymbol{O}_{\boldsymbol{2}}\boldsymbol{\to}\boldsymbol{OH}^{\boldsymbol{.}}\boldsymbol{+}\boldsymbol{O}_{\boldsymbol{2}}\boldsymbol{+}\boldsymbol{HO}_{\boldsymbol{2}}^{\boldsymbol{.}}$ **(11)**

$\boldsymbol{O}_{\boldsymbol{3}}\boldsymbol{+}\boldsymbol{H}_{\boldsymbol{2}}\boldsymbol{O}\boldsymbol{\to}\boldsymbol{H}_{\boldsymbol{2}}\boldsymbol{O}_{\boldsymbol{2}}\boldsymbol{+}\boldsymbol{O}_{\boldsymbol{2}}$ **(12)**

$\boldsymbol{h\nu+}\boldsymbol{H}_{\boldsymbol{2}}\boldsymbol{O}_{\boldsymbol{2}}\boldsymbol{\to}{\boldsymbol{2}\boldsymbol{OH}}^{\boldsymbol{.}}\boldsymbol{+ h\nu}$ **(13)**

**IV. HRMS analysis of CV and RB5**

We performed HRMS analysis for the 25 mg L^-1^ of CV and 50 mg L^-1^ of RB5 dyes solutions before and after 15 minutes of plasma treatment, as illustrated in Fig. 3 and 4, respectively. The HRMS data reveal significant degradation pathways for both dyes, highlighting the formation of various intermediates by analyzing their mass-to-charge ratios (m/z). For RB5, the untreated dye shows a molecular ion peak at m/z 877, representing its intact structure. After plasma treatment, intermediates are detected at m/z 718.63, 306.63, and 148.63, suggesting desulfonation, azo bond cleavage, and progressive fragmentation into smaller aromatic compounds. Similarly, for CV, the untreated dye exhibits a peak at m/z 372, with intermediates at m/z 331.82, 262.29, and 182.74 emerging post-treatment. These shifts indicate demethylation, hydroxylation, and aromatic ring cleavage, eventually leading to final benzene-based fragments at m/z 106.59. In both RB5 and CV degradation pathways, the ultimate products include CO₂, H₂O, carboxylic acids, and formic acid. These final products have m/z values below 100, which are not displayed in the HRMS spectra. This reflects that plasma treatment facilitates dye degradation by breaking down complex structures into smaller, less toxic intermediates, supporting the efficacy of the plasma source in environmental dye remediation.

**
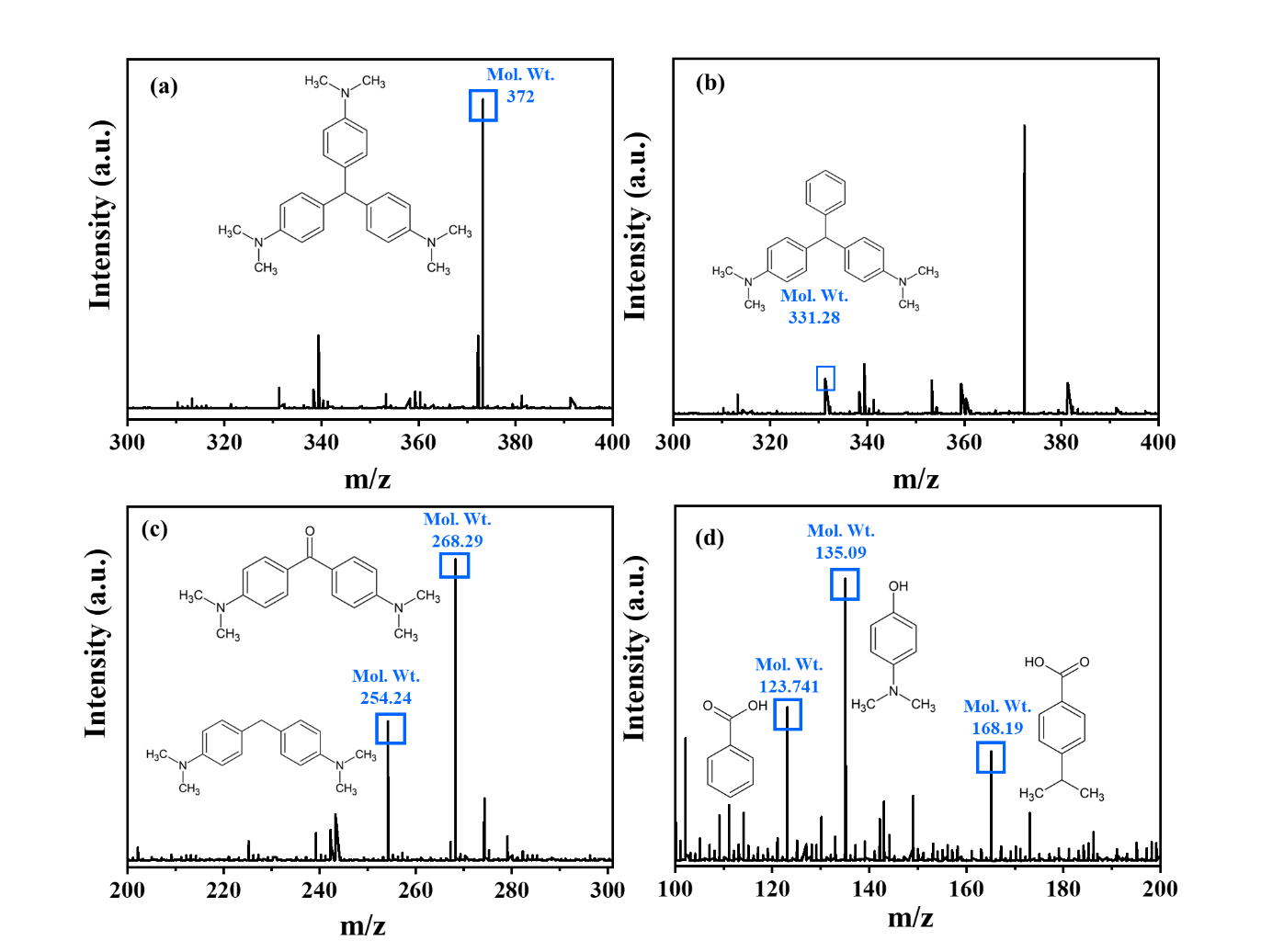
**

**Fig. 3** HRMS analysis of CV showing (a) untreated and (b, c, d) treated intermediate products.


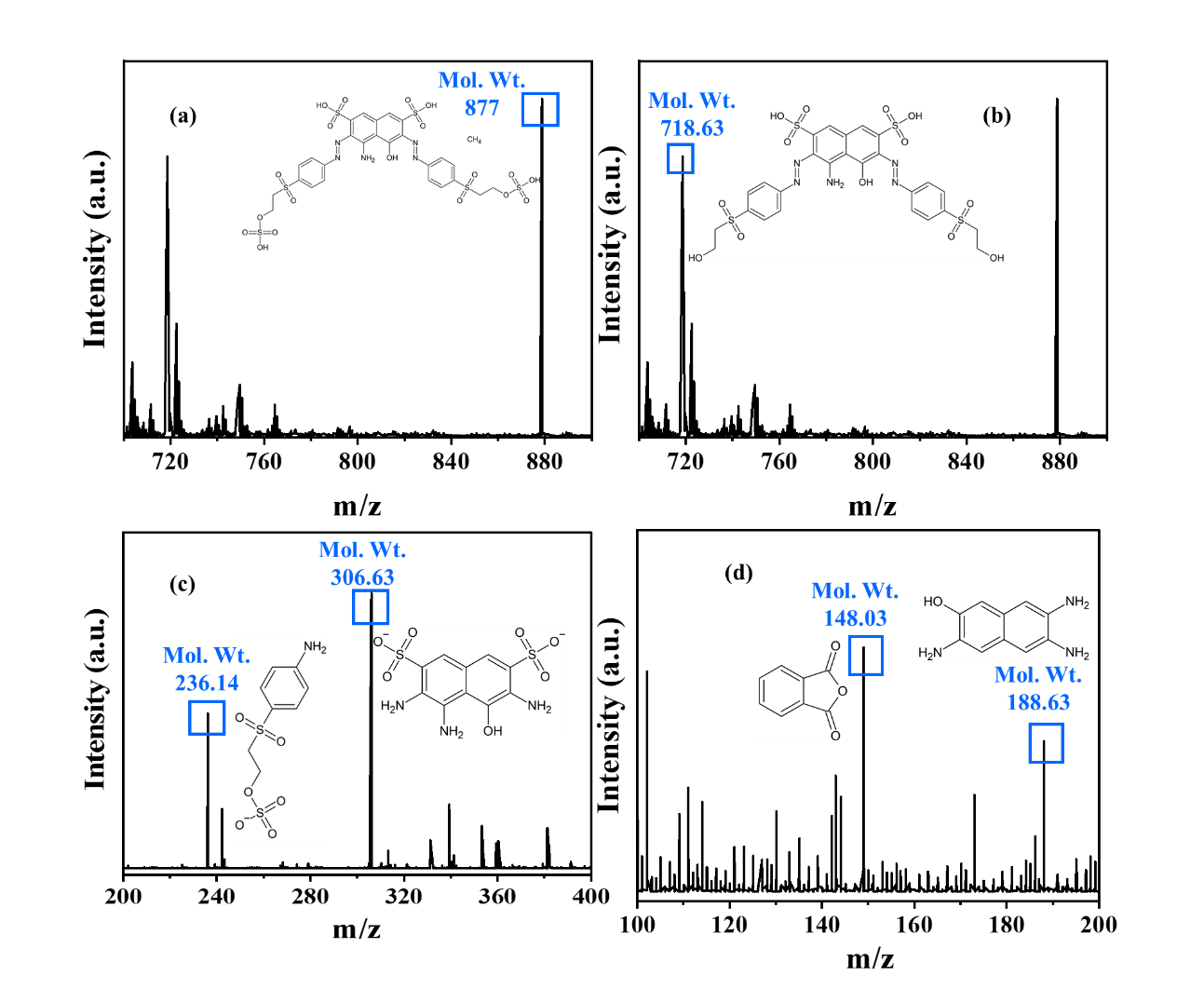


**Fig.4** HRMS analysis of RB5 showing (a) untreated and (b, c, d) treated intermediate products.

1. **Proposed degradation pathways of RB5 and CV**

The degradation of dyes like RB5 and CV involves oxidative steps initiated by reactive species at the plasma-liquid interface, as shown in Fig.5. The initial state (R₀) of RB5 is characterized by its molecular structure (C₂₆H₁₆N₆O₁₉S₆), which contains multiple sulfonate (–SO₃H) and azo (–N=N–) groups, contributing to the dye's complexity. Its molecular weight is 877. During the primary degradation step (R₁), plasma generates reactive species, such as hydroxyl radicals (•OH), which targets the RB5 molecule. These reactive species cause oxidative cleavage of the azo bonds (–N=N–) and desulfonation, reducing the molecular weight. This process results in an intermediate molecule with a molecular weight of 718 (C₂₆H₁₆N₆O₁₀S₄), marked by partial loss of sulfonate groups and cleavage of one azo bond. From this stage, the degradation of RB5 follows branching pathways. In Route 1 (R₂, R₄), further desulfonation and oxidation cause the breakdown of aromatic rings. The R₂ pathway leads to the formation of aromatic intermediates with intact sulfur groups, while the R₄ pathway results in full-ring cleavage, yielding smaller, ring-opened products with byproducts like CO₂, SO₄²⁻, and NO₃⁻. In Route 2 (R₃, R₅), the decomposition of azo bonds produces smaller aromatic amines or phenols. The R₃ pathway involves the breakage of the central naphthalene structure, while the R₅ pathway leads to continuous oxidation, eventually forming smaller aromatic fragments and ring-opening products. In the final stage, the mineralization of RB5 produces end products such as CO₂, SO₄²⁻, NO₃⁻, and H₂O. Over time, RB5 undergoes complete mineralization, breaking down into small, non-toxic molecules.

For CV, which has a triphenylmethane structure with amine groups (–N(CH₃)₂), cold plasma generates reactive oxygen species (•OH, O₂•⁻), initiating degradation through three pathways dealkylation (C₁), hydroxylation (C₂), and oxidative cleavage (C₃). As oxidation progresses, intermediates undergo di-hydroxylation (C₄) and cleavage into smaller benzene rings (C₅), eventually leading to full mineralization into CO₂, H₂O, and possibly carboxylic acids (C₇). Over time, both dyes are completely mineralized into non-toxic end products such as CO₂, sulfate (SO₄²⁻), nitrate (NO₃⁻), and water. The reduction in toxicity is confirmed through tests discussed in the section 3.8 of MS, further highlighting the efficiency of cold plasma in dye degradation and wastewater treatment.


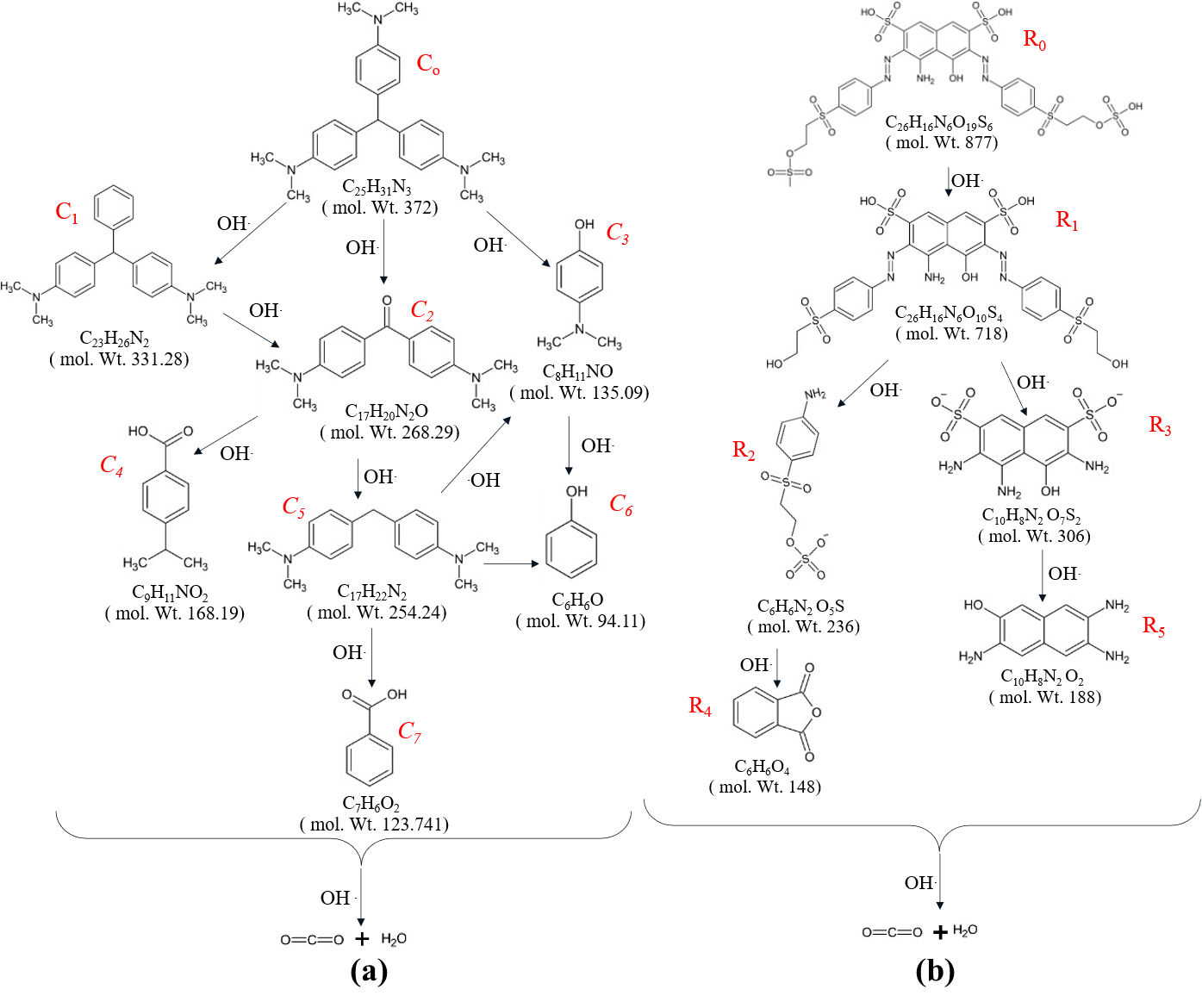


**Fig.5** Proposed degradation pathways of (a) CV and (b) RB5 using cold plasma.

1. **Cytotoxicity tests**

**Fig.6** Phase-contrast microscopy images of NIH3T3 mouse fibroblast cells (a) only cell (b) DI water and quantification of PI-positive (dead) cells using flow cytometry for (c) only cell (d) DI water.


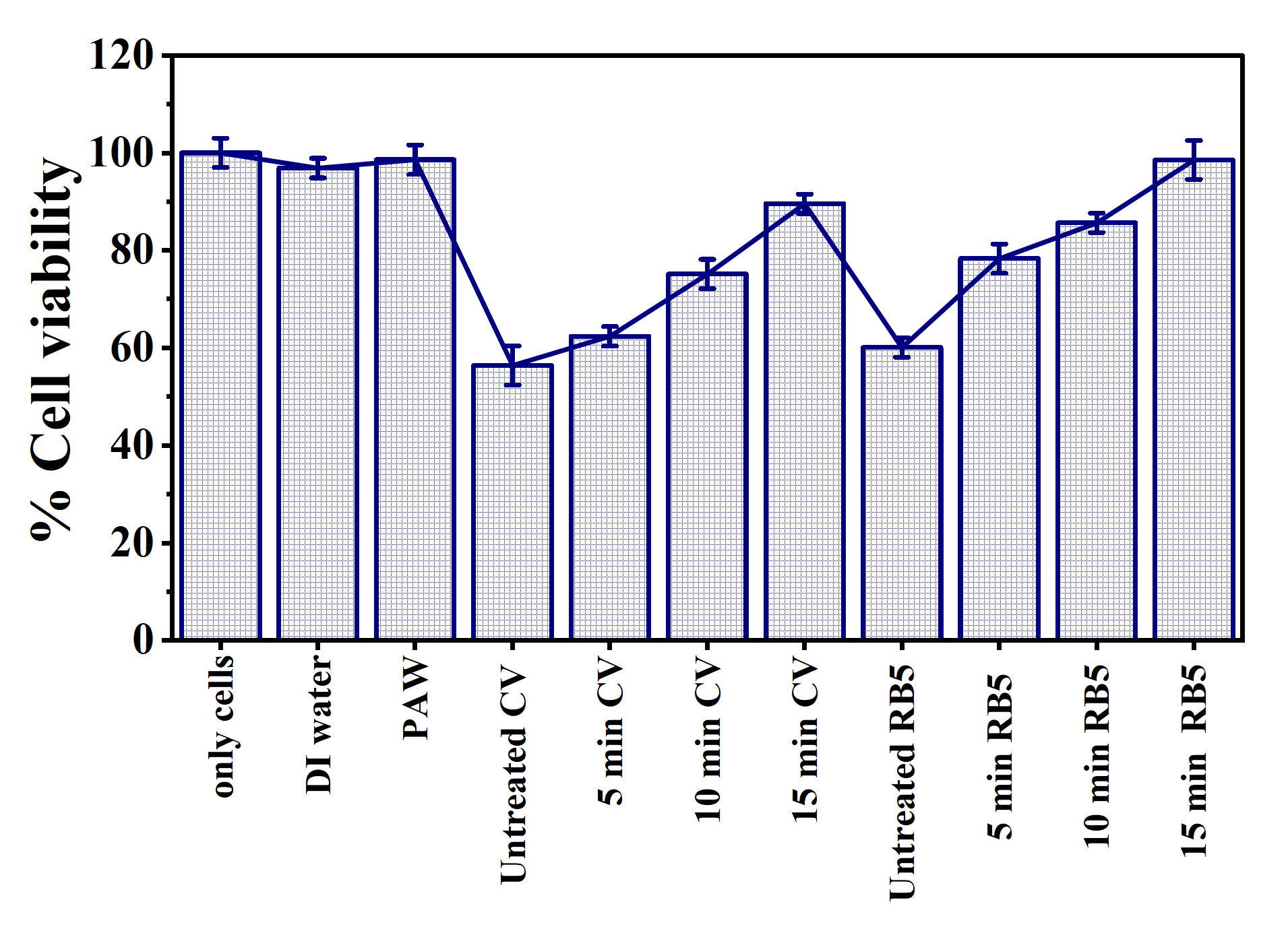


**Fig.7** Quantitative analysis of cell death in plasma-treated CV and RB5 dyes using MTT assay. For all the samples, the assay was performed in triplicate, and the data were expressed as mean ± S.D.

1. **Energy Yield Analysis**

To provide a preliminary assessment of process efficiency and practical viability, the energy yield (mg kWh⁻¹) of the P2PDBD system was estimated based on the mass of dye removed per unit electrical energy consumed and compared with previously reported plasma and advanced oxidation systems. The obtained values indicate comparatively high energy efficiency, particularly considering that the treatment was performed at relatively higher initial dye concentrations and within short treatment durations. Unlike catalytic or chemically assisted processes, the present technique operates without external reagents or catalyst materials, thereby minimizing secondary waste generation and associated handling requirements. These results suggest that the P2PDBD approach offers favorable energy utilization and holds promising potential for scalable wastewater treatment applications, although detailed pilot-scale techno-economic analyses will be required for precise cost evaluation.

| **System** | **Dyes** | **C_0_** | **P** | **η** | **TT** | **Y** | **Ref.** |
| --- | --- | --- | --- | --- | --- | --- | --- |
| Non-thermal plasma and BiPO4 | CV | 80 | 60 | 80 | 5 | 1280 | [4] |
| copper ferrite nanoparticles | CV and AR88 | 55 | **-** | 94.26 | 80 | **-** | [5] |
| Plasma jet | MB | 50 | 150 | 95 | 30 | 32 | [6] |
| Biosorbent Trifolium repens stem powder | CV | 70 | **-** | 92.99 | 140 | **-** | [7] |
| Pencil Plasma Jet | CV | 1 | 3 | 96.30 | 10 | 96.28 | [8] |
| Carbon/ZnO composite | CV | **40.80** | **-** | 94.40 | 180 | - | [9] |
| P2PDBD | RB5 | 100 | 36.2 | 94.40 | 15 | 312.93 | This work |
| P2PDBD | CV | 50 | 36.2 | 97.90 | 15 | 160.89 | This work |
| P2PDBD | CV & RB5 | 25 & 50 | 36.2 | 97.03 | 15 | 243.89 | This work |

*C_o_: Max. initial Concentration (mg/L), P: Max. discharge power (Watts), η: Max. degradation efficiency (%), Y : Energy yield (mg kWh⁻¹), TT: Max. Treatment time (min), acid red 88: AR88, crystal violet: CV & reactive black 5: RB5, Methylene blue: MB.

**References:**

1. Ahmad, R. & Ansari, K. Comparative study for adsorption of congo red and methylene blue dye on chitosan modified hybrid nanocomposite. *Process Biochemistry* **108**, 90–102 (2021).

2. Singh, R. K., Babu, V., Philip, L. & Ramanujam, S. Applicability of pulsed power technique for the degradation of methylene blue. *Journal of Water Process Engineering* **11**, 118–129 (2016).

3. Pandey, S., Mishra, R., Mishra, A., Jangra, S. & Prakash, R. Plasma activated water generation in pin-to-plate gas phase DBD-based plasma source for enhanced biochemical activity. *Phys. Lett. A* **535**, 130245 (2025).

4. Chen, J. *et al.* Non-thermal plasma and BiPO4 induced degradation of aqueous crystal violet. *Sep. Purif. Technol.* **179**, 135–144 (2017).

5. García, M. C. *et al.* Microwave atmospheric pressure plasma jets for wastewater treatment: Degradation of methylene blue as a model dye. *Chemosphere* **180**, 239–246 (2017).

6. Al-Wasidi, A. S., Abdelrahman, E. A., Rehman, K. ur, Saad, F. A. & Munshi, A. M. Efficient removal of crystal violet and acid red 88 dyes from aqueous environments using easily synthesized copper ferrite nanoparticles. *Sci. Rep.* **14**, (2024).

7. Gul, S., Afsar, S., Gul, H. & Ali, B. Removal of crystal violet dye from wastewater using low-cost biosorbent Trifolium repens stem powder. *Journal of the Iranian Chemical Society* **20**, 2781–2792 (2023).

8. Ramanathan, S. *et al.* Synergistic photocatalytic degradation of crystal violet dye using novel medical waste-derived carbon/ZnO composite: A study on toxicological assessment. *Process Safety and Environmental Protection* **187**, 145–158 (2024).

9. Rathore, V. *et al.* Optimizing Dielectric Barrier Discharge Pencil Plasma Jet Treatment for Efficient Degradation of Organic Contaminants in Denim Industry Wastewater. *Plasma Chemistry and Plasma Processing* https://doi.org/10.1007/s11090-025-10544-5 (2025) doi:10.1007/s11090-025-10544-5.
